# Emergence of Bat-Related Betacoronaviruses: Hazard and Risks

**DOI:** 10.3389/fmicb.2021.591535

**Published:** 2021-03-15

**Authors:** Roger Frutos, Jordi Serra-Cobo, Lucile Pinault, Marc Lopez Roig, Christian A. Devaux

**Affiliations:** ^1^Centre de coopération Internationale en Recherche Agronomique pour le Développement, UMR 17, Intertryp, Montpellier, France; ^2^Institut d’Électronique et des Systèmes, UMR 5214, Université de Montpellier-CNRS, Montpellier, France; ^3^Department of Evolutionary Biology, Ecology and Environmental Sciences, University of Barcelona, Biodiversity Research Institute, Barcelona, Spain; ^4^Aix Marseille University, IRD, APHM, MEPHI, IHU-Méditerranée Infection, Marseille, France; ^5^Centre National de la Recherche Scientifique, Marseille, France

**Keywords:** coronavirus, COVID-19, SARS, MERS, hazard and risks assessment

## Abstract

The current Coronavirus Disease 2019 (COVID-19) pandemic, with more than 111 million reported cases and 2,500,000 deaths worldwide (mortality rate currently estimated at 2.2%), is a stark reminder that coronaviruses (CoV)-induced diseases remain a major threat to humanity. COVID-19 is only the latest case of betacoronavirus (β-CoV) epidemics/pandemics. In the last 20 years, two deadly CoV epidemics, Severe Acute Respiratory Syndrome (SARS; fatality rate 9.6%) and Middle East Respiratory Syndrome (MERS; fatality rate 34.7%), plus the emergence of HCoV-HKU1 which causes the winter common cold (fatality rate 0.5%), were already a source of public health concern. Betacoronaviruses can also be a threat for livestock, as evidenced by the Swine Acute Diarrhea Syndrome (SADS) epizootic in pigs. These repeated outbreaks of β-CoV-induced diseases raise the question of the dynamic of propagation of this group of viruses in wildlife and human ecosystems. SARS-CoV, SARS-CoV-2, and HCoV-HKU1 emerged in Asia, strongly suggesting the existence of a regional hot spot for emergence. However, there might be other regional hot spots, as seen with MERS-CoV, which emerged in the Arabian Peninsula. β-CoVs responsible for human respiratory infections are closely related to bat-borne viruses. Bats are present worldwide and their level of infection with CoVs is very high on all continents. However, there is as yet no evidence of direct bat-to-human coronavirus infection. Transmission of β-CoV to humans is considered to occur accidentally through contact with susceptible intermediate animal species. This zoonotic emergence is a complex process involving not only bats, wildlife and natural ecosystems, but also many anthropogenic and societal aspects. Here, we try to understand why only few hot spots of β-CoV emergence have been identified despite worldwide bats and bat-borne β-CoV distribution. In this work, we analyze and compare the natural and anthropogenic environments associated with the emergence of β-CoV and outline conserved features likely to create favorable conditions for a new epidemic. We suggest monitoring South and East Africa as well as South America as these regions bring together many of the conditions that could make them future hot spots.

## Introduction

The current pandemic of Coronavirus Disease 2019 (COVID-19; Zhu et al., 2019; Guan et al., 2020), which has caused an estimated 2,500,000 deaths to date, is only the latest example that viruses sometimes leave their sylvatic environment to accidentally infect humans. The etiological agent of COVID-19, SARS-CoV-2 (Severe Acute Respiratory Syndrome Coronavirus 2), is a betacoronavirus (β-CoV; Sarbecovirus) that shares many genetic similarities with bat-borne β-CoV (Zhou P. et al., 2020; Zhou H. et al., 2020) and, to a lower extent, with β-CoV detected in Malayan pangolins, *Manis javanica* (Liu et al., 2019). Before COVID-19, two other major β-CoV-related epidemics occurred. The Severe Acute Respiratory Syndrome (SARS) in 2003, which affected mostly Guangzhou, Hong Kong, Taiwan, and Canada with exported cases around the world, and the Middle East Respiratory Syndrome (MERS) in 2012, which was limited as an outbreak in the Arabian Peninsula with cases exported to other continents. COVID-19 is a true pandemic.

Prior to 2003, when SARS emerged in China, the only coronaviruses known to infect humans were the alphacoronaviruses (α-CoV) HCoV-229E and HCoV-OC43, identified in the 1960s and responsible for the seasonal (winter) common cold. Over the past 20 years, a significant increase in the circulation of genetically related coronaviruses (CoVs) was observed. Five human outbreaks of CoVs inducing respiratory diseases were reported, including SARS-CoV (Severe Acute Respiratory Syndrome Coronavirus), HCoV-NL63, HCoV-HKU1, MERS-CoV (Middle East Respiratory syndrome Coronavirus), and SARS-CoV-2. Other outbreaks will certainly occur, although it is impossible to predict where and when this will happen. The only way to counteract this threat is to accumulate knowledge about viruses, their hosts and their dynamic of transmission, identify regions with the characteristics of potential hot-spots, and then improve regional monitoring and develop warning and intervention tools. To this end, it is important to explore the dynamic of CoVs in wildlife and the reasons for their transfer to humans.

The “One Health” concept recognizes that human health is linked to animal health and to ecosystems (Zinsstag et al., 2005). We find the source of emerging communicable diseases in our environment. CoVs (order Nidovirales, family Coronaviridae, subfamily Coronavirinae) are enveloped viruses with large plus-stranded RNA genomes of 26–32 kb. According to the International Committee of Taxonomy of Viruses (ICTV, 2020a), they were formerly classified into three genera containing viruses pathogenic for mammals (*Alphacoronavirus* and *Betacoronavirus*) and, foremost, birds (*Gammacoronavirus*). These alpha-, beta-, and gamma-coronaviruses were also referred to as CoV groups 1, 2, and 3 (Drexler et al., 2010). A new classification has recently been proposed by ICTV (2020b). According to this new classification, SARS-CoV is positioned as follows: realm Riboviria, order Nidovirales, suborder Comidovirineae, family Coronaviridae, subfamily Orthocoronavirinae, genus *Betacoronavirus*, subgenus *Sarbecovirus*, species *Severe acute respiratory syndrome-related coronavirus*, and MERS-CoV is assigned to the genus *Betacoronavirus*, subgenus *Merbecovirus.* The genus *Betacoravirus* includes the five following subgenera: *Embecovirus* (e.g, HCoV-OC43, HCoV-HKU1), *Hibecovirus* (e.g., Bat Hp-betacoronavirus Zhejiang2013), *Merbecovirus* (e.g, MERS-CoV, Pipistrellus bat coronavirus HKU5, and Tylonycteris bat coronavirus HKU4*), Nobecovirus* (e.g, *Rousettus* bat coronavirus HKU9), and *Sarbecovirus* (e.g, SARS-CoV, SARS-CoV-2 and bat SARS-batCoV HKU3). In this article, we consider and compare the different aspects involved in the three major human epidemics/pandemics of coronaviruses, i.e., SARS, MERS, and COVID-19.

## Of Bats and Coronaviruses

Bats constitute a unique group of mammals of the order *Chiroptera*, with no less than 1,230 species. Bats have been recognized as an important source of zoonotic viruses, in particular CoVs, which account for 31% of their virome (Lau et al., 2010; Chen et al., 2014; Han et al., 2015). More than 200 viruses from 28 families have been isolated or detected in bats. Bats, along with rodents, are the most important source of zoonotic viruses (Luis et al., 2013; O’Shea et al., 2014; Mollentze and Streicker, 2020). However, how to explain that bats are so much infected with CoVs? The origin of bats is estimated around 64 million years, just following the Cretaceous-Tertiary boundary (Teeling et al., 2005). The evolution of bats is a very successful singular history among mammals that led to an enormous diversity of species occupying different environments. Indeed, bats have colonized most of the terrestrial ecosystems. However, the main characteristic of bats, unique among mammals, is their ability to fly. The evolution of flight in bats seems to have selected along a unique set of antiviral immune response genes that control virus propagation, while limiting self-damaging inflammatory responses. Several adaptations have been discovered in bat cells that enable robust antiviral immune responses against RNA viruses (Subudhi et al., 2019; Banerjee et al., 2020; Hayward et al., 2020). Bats display a contracted IFN locus with only three functional IFN-α, but constitutively and permanently expressed (Zhou et al., 2016). This constitutive expression is a highly effective system for controlling viral replication that may help explain the bats’ remarkable resistance to viruses (Omatsu et al., 2007; Storm et al., 2018). Some species are migratory, a behavior that can lead to the dissemination of viruses over a large area (Colombi et al., 2019). They also display a longer lifespan than most mammal species of the same size. Some bat species are gregarious and form colonies where individuals are in close contact, which can provide opportunities for viral exchange. For example, some bat colonies have more than one thousand individuals per square meter (Serra-Cobo and López-Roig, 2016). Owing to a long coevolution between viruses and bats, an association was developed between families or genera of viruses and bat genera. The human α-CoV HCoV-229E described in the 1960s (Hamre and Procknow, 1966) was reported to share a common ancestor with Ghanaian *Hipposideros* spp. bats (Pfefferle et al., 2009). The CoVs that have emerged in humans over the last twenty years and are suspected to originate from wildlife, all belong to the β-CoV genus, either to lineage A/currently subgenera *Embecovirus* (HCoV-HKU1), lineage B/*Sarbecovirus* (SARS-CoV and SARS-CoV-2), or lineage C/*Merbecovirus* (MERS-CoV) (ICTV, 2020b). While bats and birds are considered hosts for ancestors of most CoVs, lineage A β-CoVs have not been found in these animals, but are rather considered to be related to the ChRCoV HKU24 from Chinese *Rattus* (Lau et al., 2015). Sarbecoviruses and Merbecoviruses infecting humans and related viruses have been found in bats. SARS-CoV, which emerged in humans in 2002–2003, is considered to have diverged from bat-borne CoVs only a few years before the outbreak of the epidemic (Lau et al., 2005, 2010). MERS-CoV, which emerged in 2012, has been reported to be closely related to Ty-BatCoV HKU4 borne by *Tylonycteris pachypus* and to Pi-BatCoV HKU5 borne by *Pipistrellus abramus* (Woo et al., 2007, 2012). CoV HKU4 and CoV HKU5 exhibit between 75.3 and 81.2% nucleotide identity, respectively, with MERS-related CoVs. They have been reported in *T. pachypus* and *P. pipistrellus* bats in China (Luo et al., 2018). These bat species are also found in the Arabian Peninsula, where MERS emerged. However, the only bat species in which MERS-CoV was found in the Arabian Peninsula is *Taphozous perforatus.* One specimen was captured in Bisha, Saudi Arabia, near the home of the MERS-CoV index-case patient (Memish et al., 2013). A MERS-like virus, 85% identical to MERS-CoV (but with a highly divergent spike protein gene), has also been reported in *Neoromicia capensis* from South Africa (Ithete et al., 2013). Molecular clock dating estimated that the divergence with the bat ancestor virus occurred in mid-2011 (Cotten et al., 2013). SARS-CoV-2, which emerged in 2019 in China shares 96% identity with BatCoV RaTG13 from *Rhinolophus affinis* (Zhou P. et al., 2020) and RmYN02 from *Rhinolophus malayanus* (Zhou H. et al., 2020). The only batCoV sequence ever found to be identical to that of human CoV is the RNA-dependent RNA polymerase (RdRp) gene of the MERS-CoV sequenced from *T. perforatus* in Saudi Arabia.

## A Global Distribution of Bats and Coronaviruses

**FIGURE 1:**
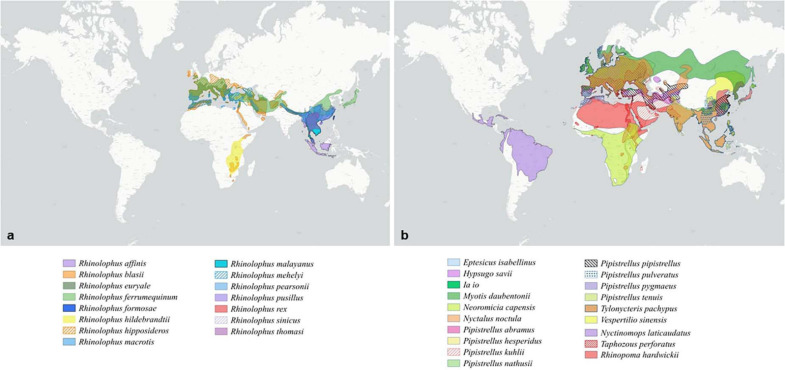
Distribution of bat species according to the group of coronavirus. **(a)** Distribution of bat species displaying an ACE2 receptor and associated with Sarbecoviruses. **(b)** Distribution of bat species displaying a DPP4 receptor and associated with Merbecoviruses.

Most studies agree that CoVs found in mammals are evolutionally linked to ancestral bat-borne coronaviruses (Hu et al., 2015). Although the accuracy of the prediction remains difficult to evaluate, it was estimated that there are at least 3,200 CoVs currently circulating in bats (Anthony et al., 2017b). Consequently, bats play an important role in the evolution of α-CoVs and β-CoVs (Drexler et al., 2014; Berto et al., 2017). A large-scale study conducted worldwide on 282 bat species belonging to 12 families showed the presence of CoVs on 8.6% of bats, whereas the ratio was only 0.2% for non-bat species (Anthony et al., 2017b). A survey conducted on 11 European bat species revealed that the diversity and prevalence of bat CoVs currently reported from Western Europe were much higher than previously thought and included a SARS-CoV sister group (Ar Gouilh et al., 2018). It is worth noting that the geographic distribution of the bat species associated with SARS-CoV, SARS-CoV-2, or MERS-CoV is not limited to the locations of disease emergence in humans. These bats are largely distributed over the old world (Figure 1A). Bats species associated with Sarbecoviruses cover Eurasia from Western Europe to East Asia, with a particularly significant prevalence in China and Continental Southeast Asia (Figure 1A). They are also, although to a lower extent, present in Sub-Saharan Africa (Figure 1A). Bat species associated with Merbecoviruses are largely present in Africa, but also in Europe and Asia (Figure 1B). Moreover, bat β-CoVs related to SARS-CoV/SAR-CoV-2 and MERS are also found in bats in different parts of the world, suggesting that the emergence of related diseases could potentially occur in locations other than China or the Arabian Peninsula. However, no outbreaks of disease have yet occurred outside of these two locations.

## A Matter of Compatibility

A key in the emergence of an infectious disease is the compatibility between the host and the virus, which is one of the three conditions for disease emergence. This compatibility is largely based on the presence of the adequate receptor for the virus on animal and human cells. Coronaviruses contain a surface-located spike, the S glycoprotein, which triggers infection by mediating receptor recognition prior to membrane fusion. Therefore, the spike protein sequence and 3-dimentional folding of this molecule are considered to determine the “host-jump” of coronaviruses (Lu et al., 2015). Human CoV, such as HCoV-OC43 and HCoV-HKU1, mainly use the histocompatibility antigen HLA Class I as a receptor (Collins, 1993; Chan et al., 2009). The HCoV-229E uses the aminopeptidase N/CD13 (Graham et al., 2013). The HCoV-NL63, SARS-CoV, and SARS-CoV-2 use the angiotensin converting enzyme 2 (ACE2), an 805 amino acid transmembrane protein, to enter human cells (Simmons et al., 2011; Graham et al., 2013; Qiu et al., 2020; Yan et al., 2020). The MERS-CoV uses the dipeptidylpeptidase 4 (DPP4)/CD26 for binding to human cells (Millet and Whittaker, 2014). In SARS-CoV, the S1 domain of the spike protein mediates ACE2-receptor attachment, a transmembrane peptidase regulating the renin-angiotensin-aldosterone system (Lambert et al., 2005; Devaux et al., 2020b). The viral receptor binding domain (RBD) was mapped into a region of the spike subdomain 1 or S1 located between the amino acid residues 318 and 510 (Babcock et al., 2004). A co-crystal structure of ACE2 and SARS-CoV spike RBD region indicated that residues 424–494 were involved in the direct interaction with the first α-helix, Lys353, and the proximal residues at the N-terminus of the β-sheet 5 of ACE2 (Li et al., 2005b). Following S1 domain binding to ACE2, the S2 domain undergoes transconformational modifications allowing membrane fusion. Most amino acid residues, essential for the SARS-CoV binding to ACE2, were conserved in the SARS-CoV-2 spike S1 domain. The structural basis for SARS-CoV2 interaction with ACE2 was also solved (Yan et al., 2020). Six RBD amino acids from the SARS-CoV S1 have been shown to be critical for binding to ACE2, namely L455, F486, Q493, S494, N501, and Y505 in SARS-CoV-2 (Andersen et al., 2020). The polymorphism of the ACE2 receptor at amino acid positions 31, 41, 82, 90, and 353 (with the most favorable residues being K31, Y41, N82, N90, K353) was used to estimate species susceptibility to SARS-CoV-2 (Devaux et al., 2021). Species such as *Macaca mulatta*, *Felis catus*, *Rhinolophus sinicus*, *M. javanica*, or *Pelodiscus sinensis* have been shown to be susceptible to SARS-CoV-2 infection. The analysis of the 3-D structures of different ACE2 receptors with respect to the amino acids found in the region 30–41, 82–93, and 353–358 was performed after designing a backbone from the *Homo sapiens* ACE2 in which the corresponding regions from *R. sinicus*, *Mus musculus*, and *Xenopus tropicalis* species were substituted to that of humans. These substitutions did not change the global 3-D structure of the molecule, but slightly altered the electrostatic pattern of the molecule with possible consequences on the affinity of the SARS-CoV-2 spike for these different ACE2 receptors. Several *in silico* studies have contributed to lengthen the list of species potentially capable of replicating the virus (Liu Z. et al., 2020; Luan et al., 2020; Qiu et al., 2020). Although *in silico* studies have the advantage of being able to quickly investigate the probability of infection among a large number of species, there is no substitute for *in vivo* experimentation. A recent study (Shi et al., 2020), investigated the *in vivo* susceptibility of animals to replicate SARS-CoV-2 and reported that the virus replicated efficiently in ferrets and cats, but poorly in dogs, pigs, chicken, and ducks. The polymorphism of ACE2 in human populations has recently been well documented (Cao et al., 2020), suggesting possible differences in human susceptibility to the virus. Based on *in silico* analyses, it was also reported that the DDP4 receptor could play a role in COVID-19 severity as a secondary receptor (Bassendine et al., 2020; Vankadari and Wilce, 2020). However, this interaction was not confirmed *in vitro* (Hoffmann et al., 2020).

**FIGURE 2:**
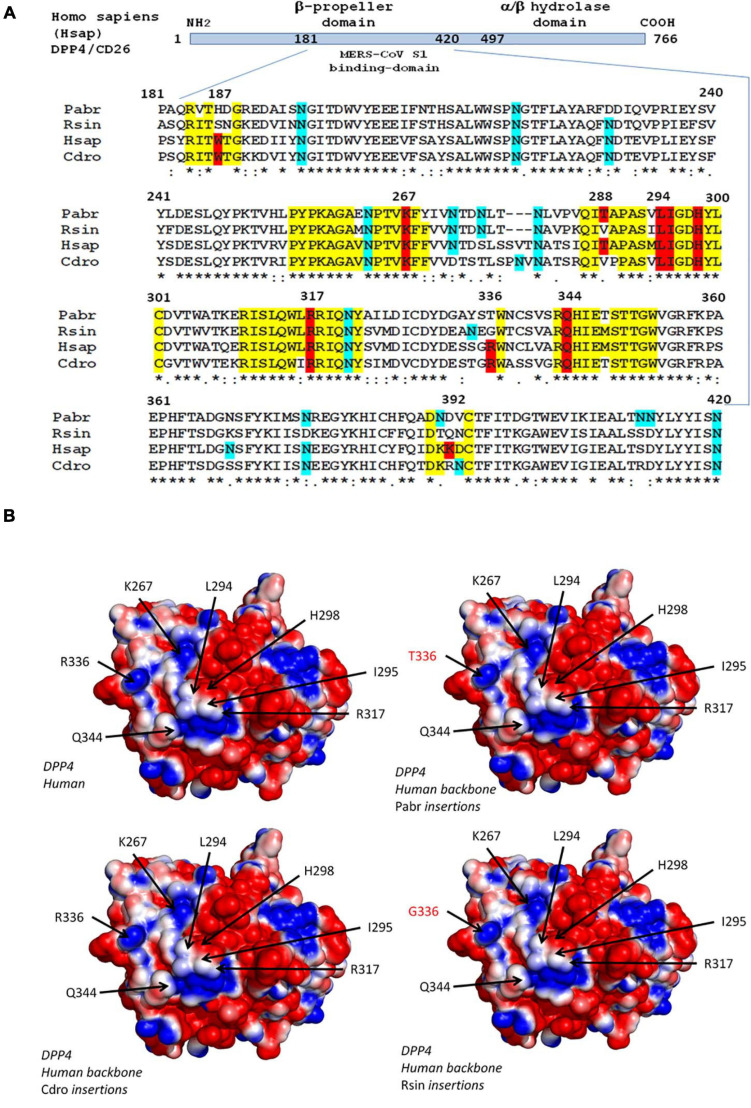
Polymorphism of the DPP4 receptor. **(A)** DPP4 multiple sequence alignments. Schematic representation of the DPP4 protein organization (upper panel). The MERS-CoV S1 spike binding site is located in a region of DPP4 that extends from amino acid position 181 to amino acid 420. A clustal omega multiple sequence alignment (EMBL-EBI bioinformatic tool; Copyright EMBL 2020), spanning the 181-420 region of DPP4 from *Pipistrella abramus* (Pabr), *Rhinolophus sinicus* (Rsin), *Homo sapiens* (Hsap), and *Camelus dromedarius* (Cdro) is shown (lower panel). All DPP4 sequences were downloaded from Genbank (NCBI): *Homo sapiens* (GenBank: AAH13329.2); *Camelus dromedarius* (GenBank: AIG55259.1); *Rhinolophus sinicus* (GenBank: AZO92863.1); *Pipistrellus abramus* (GenBank: AZO922861.1). Within the amino acid sequences of DPP4 important for MERS-CoV spike binding, the conserved amino acids are highlighted in yellow, those critical for MERS-CoV-DPP4 binding are highlighted in red and the potential N-glycosylation sites are highlighted in blue. **(B)** 3-D analysis and electrostatic potential surface potential of the DPP4 receptor. The 3-D structure of DPP4 was retrieved according to the published data (PDB : 6M1D and 4L72). Critical amino acid sequences required to allow coronaviruses spike binding to human DPP4 were substituted by the corresponding sequence from *Rhinolophus sinicus*, *Pipistrella abramus* and *Camelus dromedarius* into a human DPP4 backbone sequence to determine whether or not these substitutions may change the 3-D structure of the receptors. Protein modeling for these chimeric sequences was performed using the Phyre2 server (Kelley et al., 2015). The PyMOL 1.8.0 software (https://sourceforge. net/projects/pymol/files/pymol/1.8/) and the Adaptive Poisson-Boltzmann Solver (APBS) tools plugin (https://pymolwiki.org/index.php/APBS) was used to generate electrostatic potential surfaces of the human receptors and their modified chimeric versions. The red color indicates an excess of negative charges near the surface and the blue color arises from a positively charged surface, while white is neutral.

With respect to MERS-CoV, the viral spike binds to the DPP4/CD26 transmembrane protein on human cells (Raj et al., 2013; Wang et al., 2013). This molecule is a peptidase involved in T cell activation (Wagner et al., 2016). The structure of the MERS-CoV spike-receptor -binding domain complexed with human receptor DPP4 was deciphered (Cui et al., 2013; Wang et al., 2013). Amino acid positions 267, 291, and 346–348 are essential for virus binding. The K267E, K267N, and A291P substitutions and the Δ346–348 deletion strongly reduces binding and penetration of MERS-CoV into the target cells as well as viral replication (Kleine-Weber et al., 2020). As observed in human Sarbecoviruses, the S protein of MERS-CoV undergoes a proteolytic activation following interaction with DPP4 (Shirato et al., 2013). A multiple sequence alignment of the DPP4 sequences from 16 species is presented as Supplementary Data, which includes *H. sapiens* (Hsap); *M. mulatta* (Mmul); *Camelus dromedarius* (Cdro); *Camelus bactrianus* (Cbac); *Camelus ferus* (Cfer); *Sus scrofa* (Sscr); *Mustela putorius furo* (Mput), *F. catus* (Fcat); *Rattus rattus* (Rrat); *M. musculus* (Mmus); *P. sinensis* (Psin); *M. javanica* (Mjav); *R. sinicus* (Rsin); *Rhinolophus ferrumequinum* (Rfer); *Pipistrellus abramus* (Pabr); and *Rousettus leschenaultii* (Rles). The DPP4 protein tree was consistent with the species evolution, i.e., the DPP4 sequence from human was very close to that of *M. mulatta*, the DPP4 from the three camel species segregated together, the DPP4 from bats segregated into two groups, one comprising Psin, Tpac, and Pabr, and the other containing Rsin and Rfer (Supplementary Data). Figure 2A illustrates the polymorphism of DPP4 within the region known to be critical for MERS-CoV spike binding. Significant differences can be observed between species. The Hsap and Cdro DPP4 sequences were relatively conserved and share the critical amino acids W187, K267, L294, I295, H298, R317, H336, and Q344. However, the mutations T288V and K392R were observed. When the DPP4 from *H. sapiens* was compared to the DPP4 from *R. sinicus*, an increased diversity was found in bats with W187S, T288V, R336G, and K392Q substitutions. It could be hypothesized that the valine at position 28, common to bats and dromedaries, might have been important in the transmission of a MERS-CoV-like virus from bats to dromedary camels. The *in silico* replacement of the peptide segments 183–189, 262–269, 286–294, and 343–353 from *H. sapiens* (Hsap) by the corresponding sequences of DPP4 from *C. dromedarius* (Cdro), *P. abramus* (Pabr), and *R. sinicus* (Rsin) did not change the global 3-D structure of the molecule, but could slightly alter the electrostatic pattern of the molecule (Figure 2B). While these *in silico* studies can highlight an interspecies DPP4 polymorphism and visualize electrostatic variations that could relate to a higher or lesser susceptibility to the virus, there is a major limitation in that they only take into account the receptor component (RBD), without considering the global polymorphism of the MERS-CoV-like spikes.

## The Apparent “Multi-Host Process” of Coronavirus Emergence

An explanation for the limited number of hot-spots for coronaviruses emergence was found in the complex process of disease emergence. The presence of viruses in bats does not appear to be sufficient to trigger an epidemic in the human population. Although direct transmission has been demonstrated for some bat viruses, such as the Australian Bat Lyssavirus or the Duvenhage virus (Tignor et al., 1977; Hanna et al., 2000; Paweska et al., 2006; Afelt et al., 2018), there are currently no known cases of direct transmission of CoV from bats to humans. This led to the concept that intermediate hosts are necessary for the transfer of CoVs to humans and perhaps for humanization, i.e., adaptation to human receptors (Wu et al., 2012; Li, 2013). Both SARS-CoV and SARS-CoV-2 are considered to have originated from *Rhinolophus* bats in China (Zhou P. et al., 2020; Zhou H. et al., 2020). Indeed, Sarbecoviruses, which are very similar to SARS-CoV-2, have been described in the Chinese horseshoe bats *R. affinis* (Zhou P. et al., 2020) and *R. malayanus* (Zhou H. et al., 2020). However, no SARS-CoV was found in bats. Until now, no intermediary in the transfer of SARS-CoV and SARS-CoV-2 to humans has been formally identified. The masked palm civet, *Paguma larvata*, has been considered as intermediary for SARS (Guan et al., 2003; Watts, 2004), and the pangolin, *M. javanica*, has been accused of being responsible for the transmission of the SARS-CoV-2 to humans (Han, 2020; Liu et al., 2019; Liu Z. et al., 2020; Andersen et al., 2020), but these hypotheses have recently been contradicted (Frutos et al., 2020b). Regarding SARS-CoV-2, several animal species were considered to be possible intermediaries, i.e. susceptible hosts which can be infected and are directly involved in the transmission to humans, besides pangolin, including cat, dog, cow, buffalo, goat, sheep, swine, civet, hamster, turtle, and pigeon (Devaux et al., 2021; Luan et al., 2020; Qiu et al., 2020).

**FIGURE 3:**
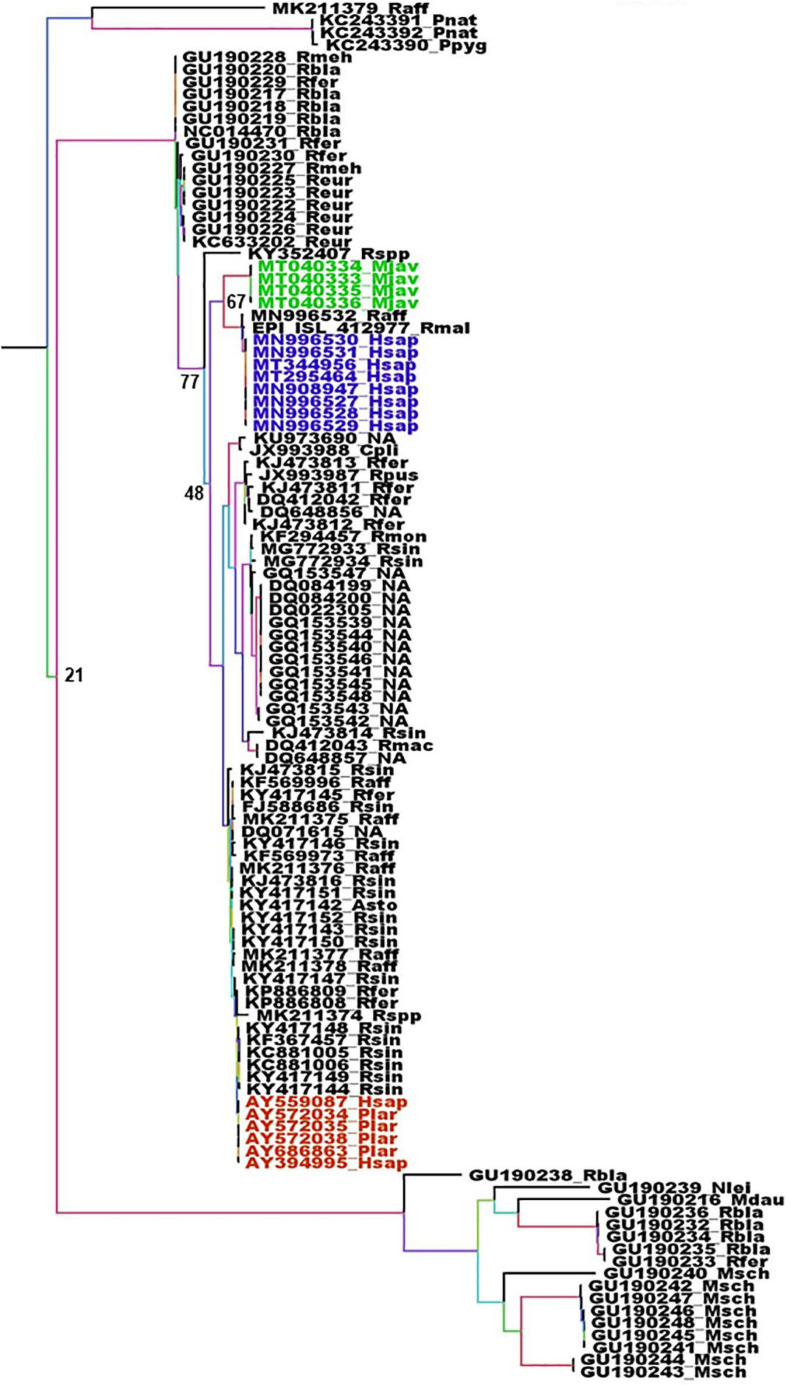
Phylogenetic analysis of Sarbecoviruses RdRp genes. The alignment of the full RdRp genes was performed with MUSCLE from the SeaView package (Gouy et al., 2010). The tree was built using the maximum likelihood method under the GTR model with 500 repeats. The tree was rooted using the RdRp sequence of a MERS-CoV from *Camelus dromedarius* (KT368883) as outgroup. Violet: RdRp sequences from human SARS-CoV-2. Green: RdRp sequences from pangolins’ Sarbecoviruses. Red: RdRp sequences from SARS-CoV. Sample names are built with the GenBank accession number followed by a four-letter code identifying the species. The species codes are as follows: Asto, *Aselliscus stoliczkanus*; Cpli, *Chaerephon plicata*; Hsap, *Homo sapiens*; Mdau, *Myotis daubentonii*; Mjav, *Manis javanica*; Msch, *Miniopterus schreibersii*; Nlei, *Nyctalus leisleri*; Plar, Paguma larvata; Pnat, *Pipistrellus nathusii*; Ppyg, *Pipistrellus pygmaeus*; Raff, *Rhinolophus affinis*; Rbla, *Rhinolophus blasii*; Reur, *Rhinolophus euryale*; Rfer, *Rhinolophus ferrumequinum*; Rmac, *Rhinophilus maculatus*; Rmal, *Rhinolophus malayanus*; Rmeh, *Rhinolophus mehelyi*; Rmon, *Rhinophilus monoceros*; Rpus, *Rhinolophus pusillus*; Rsin, *Rhinolophus sinensis*; Rspp, *Rhinolophus* unidentified species; NA, Not available.

**FIGURE 4:**
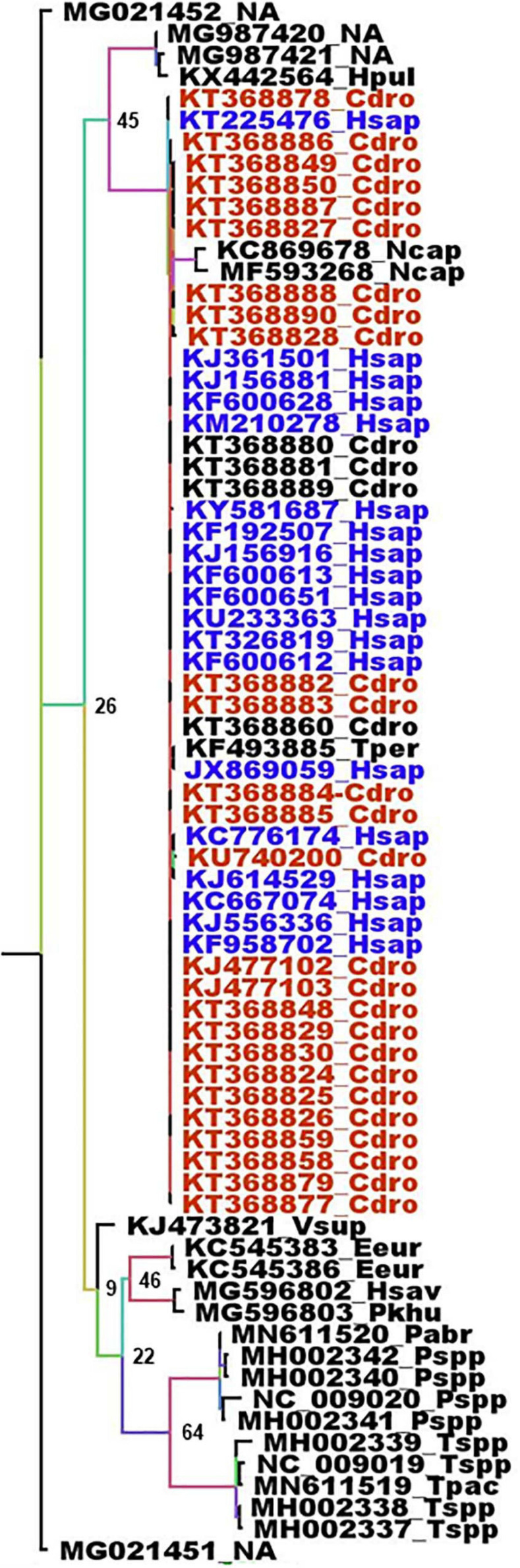
Phylogenetic analysis of Merbecoviruses RdRp genes. The alignment of the full RdRp genes was performed with MUSCLE from the SeaView package (Gouy et al., 2010). The tree was built using the maximum likelihood method under the GTR model with 500 repeats. The tree was rooted using the RdRp sequence of a Sarbecovirus from *Manis javanica* (MT040333) as outgroup. Deep blue: RdRp sequences from human MERS-CoV. Red: RdRp sequences from dromedaries MERS-CoV. Sample names are built with the GenBank accession number followed by a four-letter code identifying the species. The species codes are as follows: Cdro, *Camelus dromedarius*; Eeur, *Erinaceus europaeus*; Hpul, *Hypsugo pulveratus*; Hsap, *Homo sapiens*; Hsav, *Hypsugo savii*; Ncap, *Neoromicia capensis*; Pabr, *Pipistrellus abramus*; Pkuh, *Pipistrellus kuhlii*; Pspp, *Pipistrellus* unidentified species; Tpac, *Tylonycteris pachypus*; Tper, *Taphozous perforatus*; Tspp, *Tylonycteris* unidentified species; Vsup, *Vespertilio superans*; NA, Not available.

**FIGURE 5:**
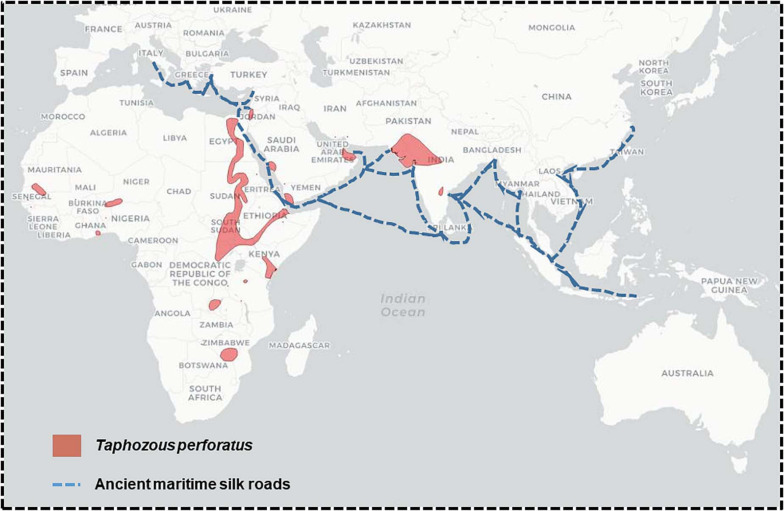
Comparison of the distribution of *Taphozous perforatus* and the ancient silk roads.

The animal intermediates, if any, for both SARS-CoV and SARS-Cov-2, still remain to be identified. The full length genome sequence of viruses circulating in wildlife could provide very valuable information regarding the origin of these viruses. Unfortunately, the number of full length CoV genomes in databases is generally limited to viruses isolated from humans, animal hosts considered as intermediaries and few bat species, usually from the region of emergence and at a time of epidemics. Most bat CoV sequences available are coming from phylogenetic studies and are thus often limited to the RdRp. Although the β-CoVs closely related to SARS_CoV and SARS-CoV-2 are mostly found in Asian bats, some circulate in European bats such as *R. ferrumequinum, Rhinolophus mehelyi, Rhinolophus blasii*, or *Rhinolophus euryale* (Figure 3). Beta-coronaviruses more distantly related are also circulating in European bats, such as *R. blasii, R. ferrumequinum, Myotis daubentonii, Miniopterus schreibersii*, or *Nyctalus leisleri* (Figure 3). The phylogenetic analysis of the RdRp gene shows two different situations with respect to SARS-CoV and SARS-CoV-2 and their respective suspected intermediaries. SARS-CoV displays the same RdRp as viruses isolated from masked palm civets, both at the gene and protein levels (Figure 3 and Supplementary Figure 1). The RdRp from SARS-CoV-2 is different from that of β-CoV from pangolin both with respect to the gene (Figure 3) and the protein (Supplementary Figure 1). Human and pangolin viruses cluster separately (Figure 3 and Supplementary Figure 1). The human SARS-CoV-2 is more closely related to the virus from the horseshoe bats *R. affinis* and *R. malayanus* (Figure 3 and Supplementary Figure 1) than to that of pangolin. Interestingly, the RdRp of the pangolin virus seems to have diverged from that of bats and human viruses (Figure 3 and Supplementary Figure 1). However, the transition/transversion ratio (Ts/Tv) displayed by these sequences is lower than 1 (0.971). This indicates that the genome is saturated, i.e., all possible mutations exist in each site, and therefore the linear correlation between time and the number of mutations is no longer valid. The consequence is that phylogeny and molecular clocks might be biased (Dávalos and Perkins, 2008; Duchêne et al., 2015). Nevertheless, the protein distance is significant and is more reliable than the gene phylogeny. In this case, both the gene and protein analyses are congruent. Although the number of sequences of the spike gene from European bats is very limited, a similar feature is found with respect to masked palm civet and pangolin. They both make separate cluster, with, in the case of SARS-CoV-2 a closer relationship with the spike gene from the *R. affinis* raTG13 than with that from pangolin (Supplementary Figure 1). These spike genomic sequences also display a Ts/Tv ratio very close to 1 (1.050) indicative of a situation very close to full genome saturation. Nevertheless, the analysis of the spike gene and protein are congruent and confirm the conclusions from the RdRp analysis, albeit with some slight differences. Both gene and protein analyses show the parallel but separate clustering of civet and human SARS-CoV with a close relationship with a virus from *R. sinicus* (Supplementary Figures 2, 3). SARS-CoV-2 from human and pangolin show the same distribution as with RdRP. They both cluster separately with the human SARS-CoV-2 being a lot closer to the virus from *R. affinis* (Supplementary Figures 2, 3). The conclusion is that both the masked palm civet and the pangolin are parallel hosts, i.e., susceptible hosts infected along with humans, but not responsible for the transmission to humans, rather than intermediaries, i.e., susceptible hosts which are infected and are directly involved in the transmission to humans, a conclusion confirming previous reports (Tu et al., 2004; Han, 2020; Liu P. et al., 2020). The circulation of human coronaviruses in other mammals is not limited to SARS-CoV and SARS-CoV-2. The human HCoV-229E shares common ancestors with alpha-CoVs from the bat *Hipposideros caffer ruber* (Pfefferle et al., 2009) and a virus infecting captive alpacas (*Vicugna pacos*), while another related virus infects camels (Corman et al., 2016). HCoV-NL63 displays sequence similarities with the bat *Perimyotis subflavus* CoV ARCoV.2, and can replicate in cell lines derived from the lungs of tricolored bats (Huynh et al., 2012). MERS-CoV is another example. MERS-CoV is closely related to both bat CoV HKU4 (found in *Tylonycteris* bats) and bat CoV HKU5 (found in *Pipistrellus* bats). The MERS epidemic was attributed to a coronavirus probably initially present in *Pipistrellus* (Wang et al., 2014; Anthony et al., 2017a) or *Taphozous* bats (Memish et al., 2013) and transmitted to humans by dromedaries, *C. dromaderius* (Azhar et al., 2014; Haagmans et al., 2014; Hemida et al., 2014; Memish et al., 2014). In the case of MERS, it is not clear whether dromedaries were intermediaries or parallel hosts. For both the RdRp and spike genes, the same sequences are found in human and dromedary viruses (Figure 4 and Supplementary Figure 4). The genome of the Merbecoviruses is also close to saturation, although the Ts/Tv ratio is slightly above 1 for both the RdRp and spike genes with values of 1.220 and 1.254, respectively. The protein tree confirms this distribution (Supplementary Figures 5, 6). However, it might be possible that humans also infect dromedaries and that a double-sense circulation may therefore exist. A MERS-CoV found in a *T. perforatus* bat in Saudi Arabia displayed the exact same RdRp sequence as a MERS-CoV from a human patient (Figure 5; Memish et al., 2013). Interestingly, the RdRp sequence of both the human and *T. perforatus* viruses slightly differ from all the other MERS-CoVs isolated from humans and dromedaries (Figure 4). No other MERS-CoV has been found in any other bat species. However, a Merbecovirus sequence amplified from the South African Bat *N. capensis* was found to be closely related to MERS-CoV based on the RdRp gene (Figure 4). When considering the spike gene and protein, other Merbecoviruses found in *Pipistrellus hesperidus* in Uganda clustered with the viruses from *N. capensis* (Supplementary Figures 6, 7).

## Emergence of a Unique Virus Population From a Metapopulation

Coronaviruses, and other viruses, are organized in metapopulations, i.e., a population of populations (Levins, 1969). A metapopulation is composed of distinct populations that share most of the genetic background, but also differ from each other for part of it. These populations cover the whole area of distribution of the metapopulation. Viruses display an additional dimension in population dispersion, since they live in other living organisms, themselves organized in metapopulations covering an area of distribution. Furthermore, coronaviruses, like other RNA viruses, display a quasispecies organization, which is host driven (Xu et al., 2004; Song et al., 2005; Tang et al., 2006; Zhang et al., 2007; Briese et al., 2014; Karamitros et al., 2020). Taken together, these populational traits give rise to a great diversity of viral populations, and therefore genotypes. The disease emergence event concerns one given population of virus among a multitude. What is described as “The virus” responsible for a disease is therefore not a microorganism selected for human pathogenesis, but only one possibility among many. The emerging virus is simply one given population of virus within a multitude of genetically related populations, i.e., a metapopulation, which by chance, hence the accidental nature of disease emergence, was exposed to the unique conjunction of biological and societal events leading to the emergence of a disease. It is very unlikely that another accidental conjunction of events will lead to the emergence of the very same single population of virus. For a given group of virus, two independent events of emergence will concern two different populations that will be very closely related since they belong to the same metapopulation, but will also be genetically distinct since they correspond to two separate populations within this metapopulation. This is what has been observed with SARS-CoV and SARS-CoV-2, causing SARS and COVID-19, respectively. Beyond coronaviruses, this is what is observed with the flaviviruses responsible for dengue. Four different dengue viruses are circulating in humans, DENV1, DENV2, DENV3, and DENV4. These viruses are simultaneously very similar and genetically and serologically different. These four viruses are resulting from four independent events of emergence in humans from the same sylvatic viral metapopulation (Moncayo et al., 2004; Vasilakis et al., 2011). There is therefore nothing exceptional in having two closely related SARS viruses that emerged a few years apart and others may emerge in the future.

## The Societal Environment of the Emergence of Coronaviruses

The circulation of CoVs involves bats but also other mammals or birds, and passages from bats to other animals might therefore be relatively frequent. Similarly, passages to humans might also be relatively frequent, but remain essentially unnoticed and rarely turn into emerging epidemics or pandemics. Most zoonotic emergences result in only few human cases and disappear with no subsequent epidemic or pandemic (Jones et al., 2008). The reason is that, beyond compatibility, another key driver for the emergence of human communicable diseases is the human societal environment, which provides the amplification loops for the outbreak to occur. The sole biological compatibility is not sufficient to trigger an outbreak. The two anthropogenic conditions required for an epidemic or pandemic to happen are contact and establishment of a human to human urban cycle. Both are consequences of human activities. The emergence of a communicable disease is only one of the many possibilities that just happen by chance due to the stochastic, unique, and unpredictable conjunction of events leading to disease emergence. The emergence of an epidemic or pandemic is a very rare event. However, it is a matter of probability and if human activities increase the frequency of anthropogenic factors leading to amplification and emergence, then the probability of occurrence will increase as well.

## A Comparative History of SARS/COVID-19 and MERS

SARS-CoV has emerged as a human disease in Guangdong, Southern China, in November 2002. The location and time of the original transmission from animal to human is not known and SARS-CoV was found in wet markets in Guangzhou and Shenzhen in masked palm civets (*P. larvata*) and raccoon dogs (*Nyctereutes procyonoides*) (Tu et al., 2004; Webster, 2004; Song et al., 2005). However, these two markets were the only places where SARS-CoV was found in small mammals. It was not found in any other masked palm civet samples from farms in Guangdong, Henan and Hunan (Tu et al., 2004). Although no virus has ever been isolated outside the two markets of Guangzhou and Shenzhen, a massive culling of civets was carried out to eradicate the “source” of infection (Tu et al., 2004; Watts, 2004). SARS-CoV-related viruses were isolated from Chinese rufous horseshoe bats *R. sinicus*. However, these viruses were not the proximal ancestors of SARS-CoV and civet viruses. Following the emergence in Guangzhou, SARS spread in the urban human population mostly around the Hong Kong bay, Taiwan, and Canada, killing 8,422 persons for a death rate of 9.6% (Chan-Yeung and Xu, 2003). Bats were designated as “reservoir” for SARS-CoV, but the exact path leading to the emergence of SARS in the human population has never been elucidated. Masked palm civets, who were pointed out at responsible for human infection, seem to simply be parallel hosts that were infected in these two markets along with humans (Tu et al., 2004; Li et al., 2005a). Furthermore, civets were successfully infected experimentally with human isolates of SARS-CoV (Li et al., 2005a; Wu et al., 2005). The virus were most likely imported to the markets from an unknown place by an unknown intermediary, which could potentially be a human being. The wet markets were not the place of origin, but simply places of amplification for SARS (Li et al., 2005a; Wu et al., 2005). Serological and molecular clock analyses indicated that SARS-CoV may have emerged 4–7 years before the reported 2003 human outbreak in China (Zheng et al., 2004; Hon et al., 2008; Bolles et al., 2011), The very same pattern was observed with SARS-CoV-2 and the associated COVID-19. COVID-19 is officially considered to have emerged in the Huanan Seafood Wholesale Market (HSWM), a wet market in Wuhan, Hubei on December 8, 2019. However, the virus had probably already been circulating since early October 2019 and the HSWM was, like the wet markets of Guangzhou and Shenzhen, a place of amplification and not the place of origin. An intermediary was proposed, the pangolin (*M. javanica*), but viruses isolated from pangolin are different from SARS-CoV-2 and indeed more distant than bat viruses (Figure 3 and Supplementary Figures 1–3). Just as for civets in the SARS-CoV case, pangolins are more likely to be parallel hosts rather than intermediaries in the transmission of SARS-CoV-2 from bats to humans (Frutos et al., 2020b). Viruses very closely related to SARS-CoV-2 have been isolated from *Rhinolophus* bats, i.e., *R. affinis* and *R. malayanus*, from Yunnan (Zhou P. et al., 2020; Zhou H. et al., 2020). However, just as for SARS-CoV, the time and location of the initial event of emergence of SARS-CoV-2 and the path from bats to humans remains unknown. In both SARS and COVID-19, the main drivers for disease emergence are human activities. A reasonable hypothesis is that the SARS-CoV-like and SARS-CoV-2-like viruses were circulating at low levels in the wild without being detected and that these viruses triggered an outbreak in densely populated cities with high population mobility after amplification in wet markets. One can expect that this pattern may reiterate in the future with the emergence of another SARS-CoV-related virus coming from the SARS metapopulation. This novel SARS-CoV-related emergence is most likely to emerge again in East Asia, partly because of its specific ecology, since the areas of distribution of several *Rhinolophus* species overlap in this region (Figure 1A), but mostly because of human activities facilitating this emergence.

Middle East Respiratory Syndrome is telling a very different story. MERS was first described as a disease on September 2012 in the Kingdom of Saudi Arabia (KSA) from a 60-year old man from the city of Bisha (Zaki et al., 2012). However, this was not the actual first case. MERS cases had been described in a hospital in Zarqa in Jordan, earlier the same year (Reusken et al., 2013a). MERS extended poorly outside the Arabian Peninsula and only through imported cases. Unlike SARS-CoV-2, and to a lower extent, SARS-CoV, there was no pandemic associated with MERS-CoV. MERS-CoV was found only in one bat sample from KSA, an Egyptian tomb bat, *T. perforatus*, originating from the vicinity of the index case in KSA (Memish et al., 2014). The only closely related MERS-like virus from bats, was found in the South African bat, *N. capensis*. MERS-CoV was detected both in dromedaries and humans throughout the Arabian Peninsula, but not in any other livestock (Reusken et al., 2013b). The major dromedary camel producers are Chad, Somalia, Sudan and Kenya, with about 7.3, 7.2, 4.8, and 3.3 million heads, respectively (Younan et al., 2016; Devaux et al., 2020a). KSA and other Arabian Peninsula countries import numerous camels from these countries (Younan et al., 2016; Devaux et al., 2020a). However, a serological survey conducted in Sudan and Qatar showed that no camel care-takers were found infected by MERS-CoV, whereas camels were indeed infected (Farag et al., 2019). Surveys have shown that dromedaries from the producing African countries have been infected with MERS-CoV (Reusken et al., 2014). Furthermore, serological analyses of archive blood samples reported the presence of MERS-CoV in dromedaries in Eastern Africa since the early 1980s (Müller et al., 2014). However, these serological analyses cannot tell whether it was the exact MERS-CoV or a related virus. Camels reared in Australia were also tested and showed no serological signs of MERS-CoV infection (Crameri et al., 2015). Camels were introduced in Australia at the end of the 19th Century, most likely from Pakistan (Crameri et al., 2015). This confirms the African origin of MERS-CoV. Interestingly, even though MERS-CoV was found in dromedaries and MERS-CoV-like viruses were identified in bats in Africa, no MERS epidemic has ever been recorded in Africa. Another interesting feature is that the only bat found infected in the Arabian Peninsula, *T. perforatus*, is an African bat which distribution outside Africa overlaps perfectly the ancient maritime silk roads (Figure 5). This suggests that contacts between dromedaries, humans and bats are very ancient and that MERS-CoV-related viruses may have widely circulated over a very long period of time.

## Hazard and Risks

MERS-CoV was found in dromedaries in the Arabian Peninsula and Africa, indicating that the MERS virus was circulating outside the Arabian Peninsula (Reusken et al., 2013b, 2014; Chu et al., 2014, 2018; Corman et al., 2014b; Müller et al., 2014). Anti MERS-CoV immunoglobulin and viral MERS-CoV RNA have been detected in camels in different African countries (Cui et al., 2013; Alagaili et al., 2014; Chu et al., 2014; Corman et al., 2014a,b; Müller et al., 2014). However, no epidemics of MERS were ever recorded in Africa, even though infected bats, dromedaries and perhaps other animals were present. No epizootics were recorded either. The same was observed with SARS. No epidemics were observed before the disease broke out in Guangdong, and no animals were found infected alongside masked palm civets and raccoon dogs in the two wet markets where the disease was detected (Song et al., 2005). Human seropositivity to SARS was evidenced on samples collected before the SARS outbreak (Zheng et al., 2004), thus indicating an early circulation of the virus in the human population without any clinical signs. The circulation of MERS-CoV and SARS-CoV long before any epidemic broke out is an indication that the mere presence of coronaviruses in wildlife and even in humans is not sufficient enough to trigger an epidemic. The danger, i.e., the presence of CoVs potentially capable of emerging as an epidemic or a pandemic is recognized. It requires human activity to amplify the frequency of virus encounters and thus create amplification loops to reach the threshold necessary to trigger an epidemic. This is where the risk lies, in the anthropogenic amplification loops. These can be assessed and the risk can then be estimated. Wet markets and human population density and mobility played a key role in the emergence of SARS (Webster, 2004; Song et al., 2005) and COVID-19 (Frutos et al., 2020a). Assessment of the risks associated with the emergence of coronaviruses can and should be carried out. However, there is a need to focus at the right level, i.e., to stop focusing solely on reaction procedures when the disease is declared, but rather to develop and implement preventive actions to block the dynamics of disease emergence before an epidemic can establish itself. This requires a in-depth analysis of the human activities in place and of how they can be modified to limit the risk of triggering an amplification loop. In East Asia, after the successive outbreaks of SARS and COVID-19, risk factors have been identified and correspond to deforestation, land-use and anthropized environments, wet markets selling wild and live animals, population density, and mobility. The amplification loop of the emergence of MERS in the Arabian Peninsula seems to be different and linked to the camel trade (Reusken et al., 2014; Younan et al., 2016; Devaux et al., 2020a).

## Sarbecoviruses: A Presence Throughout Europe

The risk of emergence of Sarbecoviruses is not limited to East-Asia. *Rhinolophus* bats harboring Sarbecoviruses closely related to SARS-CoV and SARS-CoV-2 are also found in Europe and Africa (Figures 1A, 3; Drexler et al., 2010; Ar Gouilh et al., 2018). The potential for emergence, thus the danger, exists outside Asia, as in Europe. However, what could have prevented a Sarbecovirus emergence in Europe is a difference in human activities and in the organization of society. Contacts with wildlife, live animals or animal parts occur more frequently in East Asia through wet markets, traditional pharmacopeia and farming. The societal organization in Europe is not facilitating the establishment of amplification loops as has been the case in Asia. Nevertheless, an accidental process leading to a Sarbecovirus emergence in Europe, for instance in a very specific niche like MERS in the Arabian Peninsula, cannot be excluded, even if the probability of occurrence is lower.

## MERS-CoV: A Threat for Eastern and Southern Africa

Although MERS emerged in the Arabian Peninsula, MERS-CoV is very likely to have originated in Africa. Indeed, MERS-CoV-like viruses were found in bats in South Africa and Uganda or Ghana (Annan et al., 2013; Corman et al., 2014a; Anthony et al., 2017a; Geldenhuys et al., 2018). Some of these bats are found along the camel trade road that begins in Kenya, goes up along the Nile before entering the Arabian Peninsula through Jordan and can also pass by sea transfer directly from Djibouti. The amplification loops that led to the emergence of MERS in the Arabian Peninsula did not occur in Africa. The reason might be a lower human population density in the close vicinity of camels and a lower human mobility. However, the risk of an outbreak of MERS in Africa is not negligible. The Arabian Peninsula provided a niche for the emergence of MERS, but this was due to the specific market for dromedaries in this region. Other animals, which are not found in the Arabian Peninsula but are present in Africa, could also be infected with MERS-CoV and other contacts may therefore exist beyond the camel trade. Many African countries are on track for greater development and the pressure they exert on the environment is increasing. A context similar to that of East Asia is therefore developing, which could favor the occurrence of amplifying loops in addition to the already existing contacts with wildlife. Although there are currently no reports of human cases of MERS-CoV in Eastern and Southern Africa, an emergence of MERS may well occur in the future. This conclusion is consistent with previous hypotheses suggesting that a new MERS-CoV outbreak is likely to emerge in resource-poor countries of East Africa (Anthony et al., 2017a). Africa, and in particular Eastern and Southern Africa, should be under surveillance for risk of MERS emergence with sanitary survey, and human activities should be organized to avoid the occurrence of amplification loops.

## The Amazon Rainforest and Latin America: The New Hot Spot

The ecosystem of the Amazon rainforest is favorable to the occurrence of an emerging infectious disease. A very elegant study exploring bat CoV diversity worldwide has shown that Latin America is among the hot spots to monitor (Anthony et al., 2017b). So far, there is no evidence that a CoV from a Latin American bat has been circulating among the inhabitants of this region, even at a low level, below the clinical detection threshold. Coronaviruses in South America are spreading among unrelated bat species four times less than in Africa, which could be linked, either to disparate bat species interactions in different regions, or to genetic differences in CoVs (Anthony et al., 2017b). Colombia and Venezuela are the countries with the greatest diversity of bats (Mattar and Gonzàlez, 2018). CoV RNA was found in *Carollia perspicillata* (Bt-CoV/Trinidad/1CO7B) and *Glossophaga soricina* (Bt-CoV/Trinidad/1FY2B) bats in the Caribbean island of Trinidad (Carrington et al., 2008). A bat CoV (BatCoV DR/2007) found in *Desmodus rotundus* bats in Brazil showed similarities with HCoV-OC43 (Brandao et al., 2008). In southern Brazil, where almost 40 bat species are living, an α-CoV RNA was reported in urban roosts of *Molossus molossus* and *Tadarida brasiliensis* bats (Lima et al., 2013). An α-CoV (BatCoV-*M.rufus*28/Brazil/2010) was isolated in *M. molossus* and *Molossus rufus* in Brazil and a novel β-CoV (BatCoV-*P.davyi*49/Mexico/2012) was found in *Pteronotus davyi* in Mexico, who displayed high similarities with MERS-CoV (Goes et al., 2013). A survey conducted on 606 bats from 42 species in Southern Mexico led to the identification of 9 α-CoV and 4 β-CoV, as well as another β-CoV displaying 96.5% amino acid identity with MERS-CoV in *Nyctinomops laticaudatus* (Anthony et al., 2013). The forest areas extending from Southern Mexico to Brazil, and in particular the Amazon region, represent a biodiversity hot spot largely unexplored in terms of coronaviruses diversity. Studies aiming to better understand the role of CoVs in human and animal health in Latin America, particularly in the Amazon rainforest, are very limited considering the probable risk of this region becoming a hot spot for CoV emergence. Owing to the acceleration of anthropization of the environment, in particular massive deforestation, land conversion for agriculture and mining and the creation of roads in the Amazonian rainforest, the risk of emergence of new CoVs (or other viral species)-related diseases in Latin America has increased sharply recently. More contacts with a still largely unknown fauna and virus diversity can be expected, while the development of human dwellings is increasing. There is an urgent need to closely monitor this biodiversity, but more importantly to monitor human activities and potential amplification loops. Unfortunately, monitoring the latter in this region might prove very difficult owing to the lack of coverage, the occurrence of armed conflicts and the presence of numerous illegal activities. Latin America is probably the region most at risk of hosting a future emergence of a virus epidemic.

## Conclusion

The emergence of a novel pandemic is unpredictable, although specific geographical regions, or interfaces between wildlife, livestock and humans have been identified as the source of recent emerging infectious diseases (Morse et al., 2012). However, analysis of past disease emergence events, pathogen transmission dynamics and ecosystems can help to better understand the mechanisms of disease emergence. In this study, we report evidence that coronavirus-carrying bat species are found across the planet. It could therefore be hypothesized that epidemics of coronaviruses could theoretically emerge anywhere. However, the history of epidemics such as SARS, COVID-19, and MERS demonstrates that this is not the case and that these disease outbreaks occur at the level of regional hot spots. Several conditions are essential to trigger the emergence of a disease, in particular the circulation of the virus, the ability of the virus to recognize a receptor on human cells, contacts between viruses and humans, and amplification of the phenomenon by inter-human transmission. Changes in ecosystems due to anthropization of the environment may push regions that until now have been free from coronavirus emergence to become hot spots. The global economy favors the circulation of hosts, vectors and viruses altogether. This is true for coronaviruses, with SARS in 2003, MERS in 2012 and COVID-19 in 2019, but also for other human pathogens (e.g., Dengue, Chikungunya and Zika viruses in the last decade or earlier with the pandemic (H1N1) 2009 influenza). Although the risks are known, we prefer to wait for the emergence of a disease and find culprits such as bats, pangolins or mosquitoes, rather than recognize that it is the impact of human activities on ecosystems (anthropization) and our model of global economy that are the real drivers of disease emergence.

The recent COVID-19 pandemic has created an unprecedented panic reaction among governments and populations worldwide, triggering almost global lockdowns, restrictions on people’s life and unprecedented damage to the economy. This pandemic mostly highlighted the lack of preparedness to face such a public health issue, inadequate risk assessment regarding the urgency of the situation, late reporting of early cases, with insufficient internationally coordinated actions and with detection and containment strategies, often very variable from one country to another (Peeri et al., 2020). Our global model of society today is the society of the immediate. We live in an ultra-connected world, overflown with data and fast information flows (sometimes fake news) and with a culture of reaction instead of preparation. In medicine, every effort and organization is aimed at finding a cure for a communicable disease and ultimately a vaccine, but very little is done to prevent the disease. The real challenge for the future is to be able to intervene before the step of amplification in the human population.

Viruses circulate and our modern global society, characterized by extensive movements of goods and people, greatly facilitates this circulation. However, the dissemination of coronaviruses from one region to another, as seen with COVID-19 and, to a lower extent with SARS, is not the only source of danger. Sarbecoviruses and Merbecoviruses circulate in bats and most likely in other animals, certainly in dromedaries for MERS-CoV, and perhaps also humans, outside the initial region of epidemics. Epidemics broke out in specific regions because all the elements of the accidental chain leading to the emergence of the disease were present at the time of the outbreak. Owing to specific societal traits, the probability might still be higher in these regions to see another emergence of a closely related disease in the future, since human activities are the main drivers of transmissible diseases emergence. However, since the emergence of such diseases is an accidental process, this may very well happen elsewhere, even if the probability is lower. It just takes an unfortunate conjunction of events. The emergence of a SARS-related disease is possible in Europe and, similarly, the emergence of a MERS-related disease is possible in Africa. MERS-CoV could also emerge in Asia, since MERS-CoV-like viruses (e.g., BtCoV/Ii/GD/2014-422) that have acquired a S1 spike RBD conferring the capacity to bind DPP4 receptor were found in South China vespertilionid (*Vespertilio superans*) bats (Luo et al., 2018). A MERS-CoV-like coronavirus showing 72% nucleotide identity with MERS-CoV was also isolated in *P. davyi* bats (BatCoV-*P.davyi*49/Mexico/2012) in Mexico (Goes et al., 2013). Other coronaviruses than MERS-CoV and SARS-CoV might also emerge in slightly different contexts. A coronavirus, or another kind of virus, might also emerge in South America, especially in the Amazon region. The extensive deforestation under way in the Amazon is increasing the risk of new epidemics as deforestation and anthropized environments are key drivers for the emergence of communicable diseases (Afelt et al., 2017, 2018).

Although SARS-CoV-2 has, per today, killed 2.5 million people worldwide, its mortality rate is relatively low and is currently estimated at 2.2 %. However, it is most likely in the range of 0.5–1%, as a large number of cases of infection remain asymptomatic and underestimated. MERS-CoV did not spread worldwide (2,494 cases reported), but its mortality rate was high, with an estimated rate of 34.4% (858 deaths) (WHO, 2020). What will happen if the next CoV to emerge is both very virulent and highly transmissible? It is essential that we change the way we address the risk of epidemics and pandemics. Waiting for a disease to emerge makes it more difficult to react. Preventive actions should be taken. There are conserved features between emergence events of related viruses. Coronaviruses are circulating widely, but human activities are the main drivers. These activities should therefore be organized to limit the risk of zoonotic emergence. Contacts with wildlife should also be limited. It is a priority that the uncontrolled high-scale process of deforestation, land conversion and wildlife trafficking should be stopped. The hazard is here. We now need to think about how to manage risk and how to integrate it into public health policies and international regulations.

## Author Contributions

All authors jointly proposed the idea and designed the study, performed the literature search, collected data for a specific section of the manuscript each, participated in the writing and correction of the manuscript, as well as in the production of the figures, and contributed equally to the manuscript.

## Conflict of Interest

The authors declare that the research was conducted in the absence of any commercial or financial relationships that could be construed as a potential conflict of interest.

## Supplementary Material

Supplementary Figure 1 Distribution of Sarbecoviruses RdRp protein sequences. The alignment of the translated full RdRp genes was performed with MUSCLE from the SeaView package (Gouy et al., 2010). The tree was built using the maximum likelihood method under the LG model with 500 repeats. The tree was rooted using the translated RdRp sequence of a MERS-CoV from *Camelus dromedarius* (KT368883) as outgroup. Violet: RdRp protein sequences from human SARS-CoV-2. Green: RdRp protein sequences from pangolins’ Sarbecoviruses. Red: RdRp protein sequences from SARS-CoV. Sample names are built with the GenBank accession number followed by a four-letter code identifying the species. The species codes are as follows: Asto, *Aselliscus stoliczkanus;* Cpli, *Chaerephon plicata*; Hsap, *Homo sapiens*; Mdau, *Myotis daubentonii*; Mjav, *Manis javanica*; Msch, *Miniopterus schreibersii*; Nlei, *Nyctalus leisleri*; Plar, Paguma larvata; Pnat, *Pipistrellus nathusii*; Ppyg, *Pipistrellus pygmaeus*; Raff, *Rhinolophus affinis*; Rbla, *Rhinolophus blasii*; Reur, *Rhinolophus euryale*; Rfer, *Rhinolophus ferrumequinum*; Rmac, *Rhinophilus maculatus*; Rmal, *Rhinolophus malayanus*; Rmeh, *Rhinolophus mehelyi*; Rmon, *Rhinophilus monoceros*; Rpus, *Rhinolophus pusillus*; Rsin, *Rhinolophus sinensis*; Rspp, *Rhinolophus* unidentified species; NA, Not available.

Supplementary Figure 2 Phylogenetic analysis of Sarbecoviruses spike genes. The alignment of the full spike genes was performed with MUSCLE from the SeaView package (Gouy et al., 2010). The tree was built using the maximum likelihood method under the GTR model with 500 repeats. The tree was rooted using the spike sequence of a MERS-CoV from *Camelus dromedarius* (KT368860) as outgroup. Violet: spike sequences from human SARS-CoV-2. Green: spike sequences from pangolins’ Sarbecoviruses. Red: spike sequences from SARS-CoV. Sample names are built with the GenBank accession number followed by a four-letter code identifying the species. The species codes are the following: Asto, *Aselliscus stoliczkanus*; Cpli, *Chaerephon plicata*; Hsap, *Homo sapiens*; Mjav, *Manis javanica*; Msch, *Miniopterus schreibersii*; Plar, Paguma larvata; Pnat, *Pipistrellus nathusii*; Ppyg, *Pipistrellus pygmaeus*; Raff, *Rhinolophus affinis*; Rbla, *Rhinolophus blasii*; Reur, *Rhinolophus euryale*; Rfer, *Rhinolophus ferrumequinum*; Rmac, *Rhinophilus maculatus*; Rmal, *Rhinolophus malayanus*; Rmon, *Rhinophilus monoceros*; Rpus, *Rhinolophus pusillus*; Rsin, *Rhinolophus sinensis*; Rspp, *Rhinolophus* unidentified species; NA, Not available.

Supplementary Figure 3 Distribution of Sarbecoviruses spike protein sequences. The alignment of the translated full spike genes was performed with MUSCLE from the SeaView package (Gouy et al., 2010). The tree was built using the maximum likelihood method under the LG model with 500 repeats. The tree was rooted using the translated spike gene sequence of a MERS-CoV from *Camelus dromedarius* (KT368860) as outgroup. Violet: spike protein sequences from human SARS-CoV-2. Green: spike protein sequences from pangolins’ Sarbecoviruses. Red: spike protein sequences from SARS-CoV. Sample names are built with the GenBank accession number followed by a four-letter code identifying the species. The species codes are the following: Asto, *Aselliscus stoliczkanus*; Cpli, *Chaerephon plicata*; Hsap, *Homo sapiens*; Mdau, *Myotis daubentonii*; Mjav, *Manis javanica*; Msch, *Miniopterus schreibersii*; Nlei, *Nyctalus leisleri*; Plar, Paguma larvata; Pnat, *Pipistrellus nathusii*; Ppyg, *Pipistrellus pygmaeus*; Raff, *Rhinolophus affinis*; Rbla, *Rhinolophus blasii*; Reur, *Rhinolophus euryale*; Rfer, *Rhinolophus ferrumequinum*; Rmac, *Rhinophilus maculatus*; Rmal, *Rhinolophus malayanus*; Rmeh, *Rhinolophus mehelyi*; Rmon, *Rhinophilus monoceros*; Rpus, *Rhinolophus pusillus*; Rsin, *Rhinolophus sinensis*; Rspp, *Rhinolophus* unidentified species; NA, Not available.

Supplementary Figure 4 Distribution of Merbecoviruses RdRp protein sequences. The alignment of the translated full RdRp genes was performed with MUSCLE from the SeaView package (Gouy et al., 2010). The tree was built using the maximum likelihood method under the LG model with 500 repeats. The tree was rooted using the translated RdRp sequence of a Sarbecovirus from *Manis javanica* (MT040333) as outgroup. Deep blue: RdRp protein sequences from human MERS-CoV. Red: RdRp protein sequences from dromedaries MERS-CoV. Sample names are built with the GenBank accession number followed by a four-letter code identifying the species. The species codes are the following: Cdro, *Camelus dromedarius*; Eeur, *Erinaceus europaeus*; Hpul, *Hypsugo pulveratus*; Hsap, *Homo sapiens*; Hsav, *Hypsugo savii*; Ncap, *Neoromicia capensis*; Pabr, *Pipistrellus abramus*; Pkuh, *Pipistrellus kuhlii*; Pspp, *Pipistrellus* unidentified species; Tpac, *Tylonycteris pachypus*; Tper, *Taphozous perforatus*; Tspp, *Tylonycteris* unidentified species; Vsup, *Vespertilio superans*; NA, Not available.

Supplementary Figure 5 Phylogenetic analysis of Merbecoviruses spike genes. The alignment of the full spike genes was performed with MUSCLE from the SeaView package (Gouy et al., 2010). The tree was built using the maximum likelihood method under the GTR model with 500 repeats. The tree was rooted using the spike gene sequence of a Sarbecovirus from *Manis javanica* (MT040335) as outgroup. Deep blue: spike gene sequences from human MERS-CoV. Red: spike gene sequences from dromedaries MERS-CoV. Sample names are built with the GenBank accession number followed by a four-letter code identifying the species. The species codes are the following: Cdro, *Camelus dromedarius*; Eeur, *Erinaceus europaeus*; Hpul, *Hypsugo pulveratus*; Hsap, *Homo sapiens*; Hsav, *Hypsugo savii*; Ncap, *Neoromicia capensis*; *Nspp*, *Nycteris* unidentified species; Nzul, *Neoromicia zuluensis* (synonymous to *Neoromicia capensis*); Pabr, *Pipistrellus abramus*; Phes, *Pipistrellus hesperidus*; Pkuh, *Pipistrellus kuhlii*; Pnat, *Pipistrellus nathusii*; Ppyg, *Pipistrellus pygmaeus*; Pspp, *Pipistrellus* unidentified species; Tpac, *Tylonycteris pachypus*; Tspp, *Tylonycteris* unidentified species; Vsin, *Vespertilio sinensis*; Vsup, *Vespertilio superans*; NA, Not available.

Supplementary Figure 6 Distribution of Merbecoviruses spike protein sequences. The alignment of the translated full spike genes was performed with MUSCLE from the SeaView package (Gouy et al., 2010). The tree was built using the maximum likelihood method under the LG model with 500 repeats. The tree was rooted using the spike protein sequence of a Sarbecovirus from *Manis javanica* (MT040335) as outgroup. Deep blue: spike protein sequences from human MERS-CoV. Red: spike protein sequences from dromedaries MERS-CoV. Sample names are built with the GenBank accession number followed by a four-letter code identifying the species. The species codesare the following: Cdro, *Camelus dromedarius*; Eeur, *Erinaceus europaeus*; Hpul, *Hypsugo pulveratus*; Hsap, *Homo sapiens*; Hsav, *Hypsugo savii*; Ncap, *Neoromicia capensis*; *Nspp*, *Nycteris* unidentified species; Nzul, *Neoromicia zuluensis* (synonymous to *Neoromicia capensis*); Pabr, *Pipistrellus abramus*; Phes, *Pipistrellus hesperidus*; Pkuh, *Pipistrellus kuhliiv;* Pnat, *Pipistrellus nathusii*; Ppyg, *Pipistrellus pygmaeus*; Pspp, *Pipistrellus* unidentified species; Tpac, *Tylonycteris pachypus*; Tspp, *Tylonycteris* unidentified species; Vsin, *Vespertilio sinensis*; Vsup, *Vespertilio superans*; NA, Not available.
