# Supplementary figures and images for: Emergence of Bat-Related Betacoronaviruses: Hazard and Risks
Source: Front Microbiol. 2021 Mar 15;12:591535. doi: 10.3389/fmicb.2021.591535 (PMC8005542; doi:10.3389/fmicb.2021.591535)

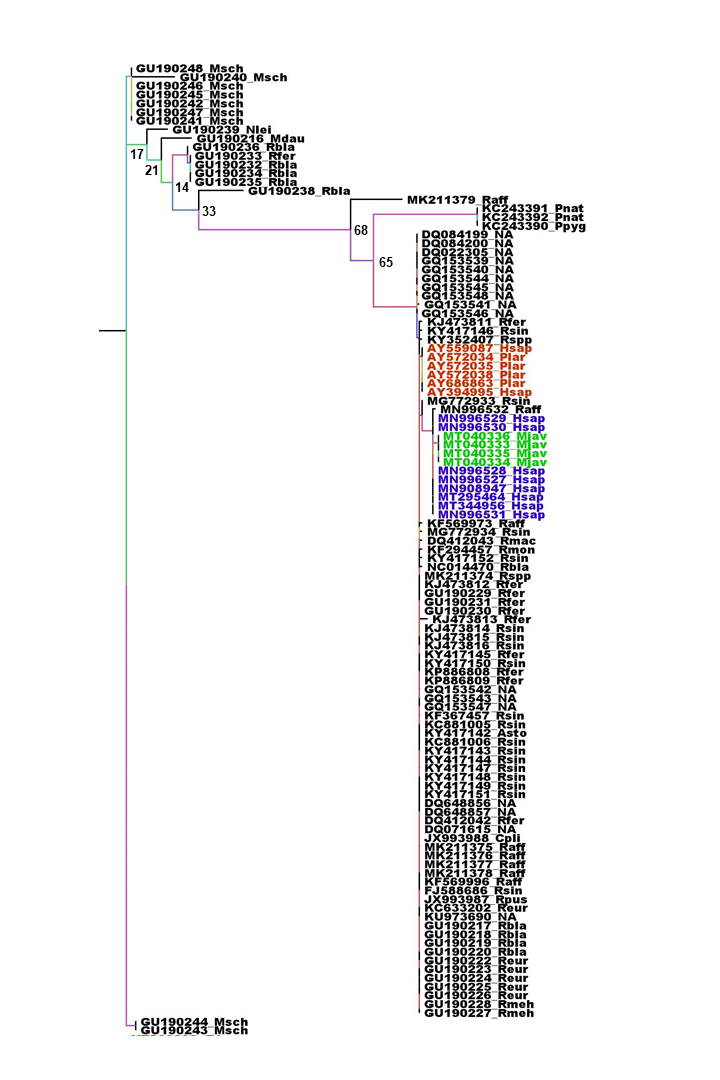

Supplement: Supplementary Figure 1 — Distribution of Sarbecoviruses RdRp protein sequences. The alignment of the translated full RdRp genes was performed with MUSCLE from the SeaView package (Gouy et al., 2010). The tree was built using the maximum likelihood method under the LG model with 500 repeats. The tree was rooted using the translated RdRp sequence of a MERS-CoV from Camelus dromedarius (KT368883) as outgroup. Violet: RdRp protein sequences from human SARS-CoV-2. Green: RdRp protein sequences from pangolins’ Sarbecoviruses. Red: RdRp protein sequences from SARS-CoV. Sample names are built with the GenBank accession number followed by a four-letter code identifying the species. The species codes are as follows: Asto, Aselliscus stoliczkanus; Cpli, Chaerephon plicata; Hsap, Homo sapiens; Mdau, Myotis daubentonii; Mjav, Manis javanica; Msch, Miniopterus schreibersii; Nlei, Nyctalus leisleri; Plar, Paguma larvata; Pnat, Pipistrellus nathusii; Ppyg, Pipistrellus pygmaeus; Raff, Rhinolophus affinis; Rbla, Rhinolophus blasii; Reur, Rhinolophus euryale; Rfer, Rhinolophus ferrumequinum; Rmac, Rhinophilus maculatus; Rmal, Rhinolophus malayanus; Rmeh, Rhinolophus mehelyi; Rmon, Rhinophilus monoceros; Rpus, Rhinolophus pusillus; Rsin, Rhinolophus sinensis; Rspp, Rhinolophus unidentified species; NA, Not available. [file Image_1.TIF]

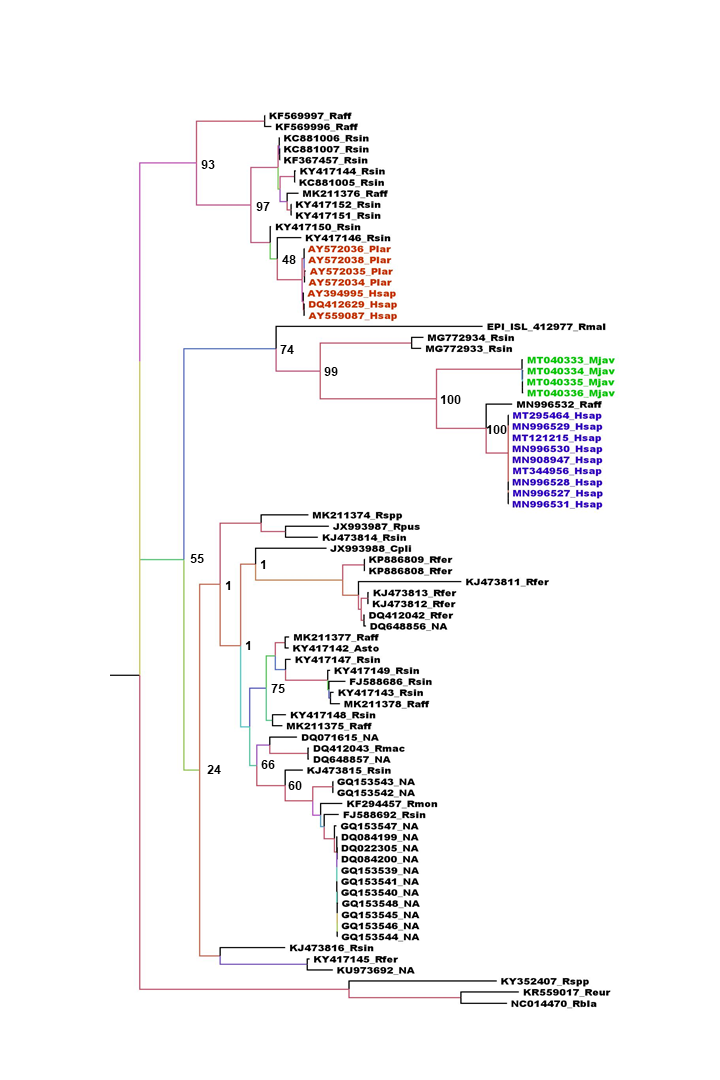

Supplement: Supplementary Figure 2 — Phylogenetic analysis of Sarbecoviruses spike genes. The alignment of the full spike genes was performed with MUSCLE from the SeaView package (Gouy et al., 2010). The tree was built using the maximum likelihood method under the GTR model with 500 repeats. The tree was rooted using the spike sequence of a MERS-CoV from Camelus dromedarius (KT368860) as outgroup. Violet: spike sequences from human SARS-CoV-2. Green: spike sequences from pangolins’ Sarbecoviruses. Red: spike sequences from SARS-CoV. Sample names are built with the GenBank accession number followed by a four-letter code identifying the species. The species codes are the following: Asto, Aselliscus stoliczkanus; Cpli, Chaerephon plicata; Hsap, Homo sapiens; Mjav, Manis javanica; Msch, Miniopterus schreibersii; Plar, Paguma larvata; Pnat, Pipistrellus nathusii; Ppyg, Pipistrellus pygmaeus; Raff, Rhinolophus affinis; Rbla, Rhinolophus blasii; Reur, Rhinolophus euryale; Rfer, Rhinolophus ferrumequinum; Rmac, Rhinophilus maculatus; Rmal, Rhinolophus malayanus; Rmon, Rhinophilus monoceros; Rpus, Rhinolophus pusillus; Rsin, Rhinolophus sinensis; Rspp, Rhinolophus unidentified species; NA, Not available. [file Image_2.TIF]

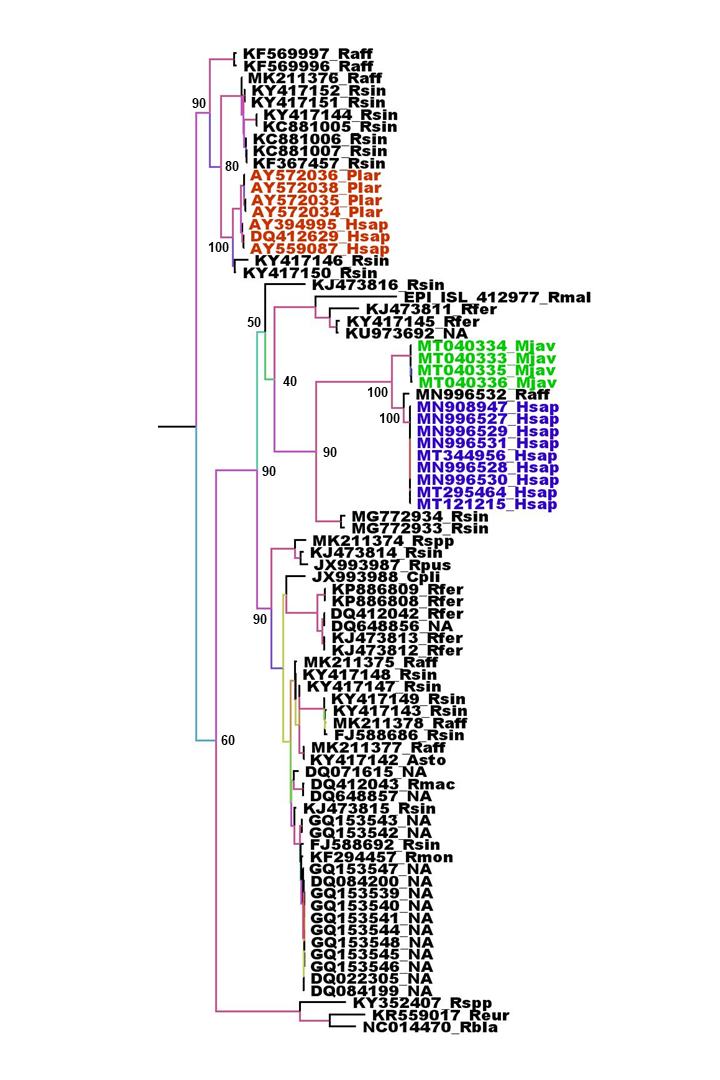

Supplement: Supplementary Figure 3 — Distribution of Sarbecoviruses spike protein sequences. The alignment of the translated full spike genes was performed with MUSCLE from the SeaView package (Gouy et al., 2010). The tree was built using the maximum likelihood method under the LG model with 500 repeats. The tree was rooted using the translated spike gene sequence of a MERS-CoV from Camelus dromedarius (KT368860) as outgroup. Violet: spike protein sequences from human SARS-CoV-2. Green: spike protein sequences from pangolins’ Sarbecoviruses. Red: spike protein sequences from SARS-CoV. Sample names are built with the GenBank accession number followed by a four-letter code identifying the species. The species codes are the following: Asto, Aselliscus stoliczkanus; Cpli, Chaerephon plicata; Hsap, Homo sapiens; Mdau, Myotis daubentonii; Mjav, Manis javanica; Msch, Miniopterus schreibersii; Nlei, Nyctalus leisleri; Plar, Paguma larvata; Pnat, Pipistrellus nathusii; Ppyg, Pipistrellus pygmaeus; Raff, Rhinolophus affinis; Rbla, Rhinolophus blasii; Reur, Rhinolophus euryale; Rfer, Rhinolophus ferrumequinum; Rmac, Rhinophilus maculatus; Rmal, Rhinolophus malayanus; Rmeh, Rhinolophus mehelyi; Rmon, Rhinophilus monoceros; Rpus, Rhinolophus pusillus; Rsin, Rhinolophus sinensis; Rspp, Rhinolophus unidentified species; NA, Not available. [file Image_3.TIF]

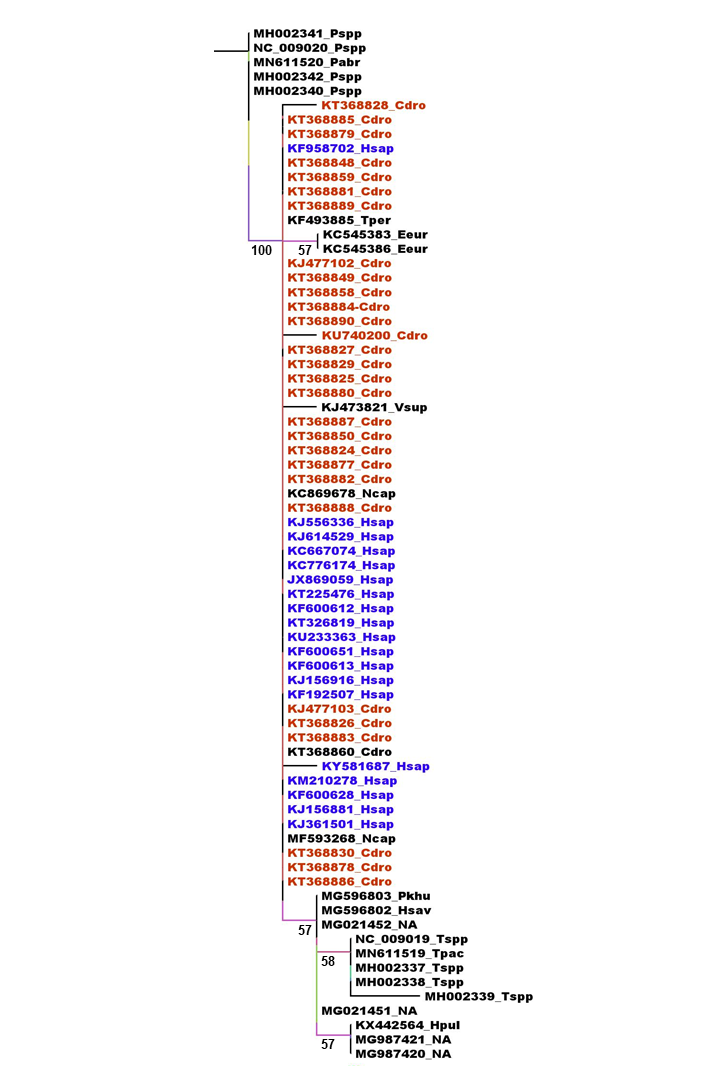

Supplement: Supplementary Figure 4 — Distribution of Merbecoviruses RdRp protein sequences. The alignment of the translated full RdRp genes was performed with MUSCLE from the SeaView package (Gouy et al., 2010). The tree was built using the maximum likelihood method under the LG model with 500 repeats. The tree was rooted using the translated RdRp sequence of a Sarbecovirus from Manis javanica (MT040333) as outgroup. Deep blue: RdRp protein sequences from human MERS-CoV. Red: RdRp protein sequences from dromedaries MERS-CoV. Sample names are built with the GenBank accession number followed by a four-letter code identifying the species. The species codes are the following: Cdro, Camelus dromedarius; Eeur, Erinaceus europaeus; Hpul, Hypsugo pulveratus; Hsap, Homo sapiens; Hsav, Hypsugo savii; Ncap, Neoromicia capensis; Pabr, Pipistrellus abramus; Pkuh, Pipistrellus kuhlii; Pspp, Pipistrellus unidentified species; Tpac, Tylonycteris pachypus; Tper, Taphozous perforatus; Tspp, Tylonycteris unidentified species; Vsup, Vespertilio superans; NA, Not available. [file Image_4.TIF]

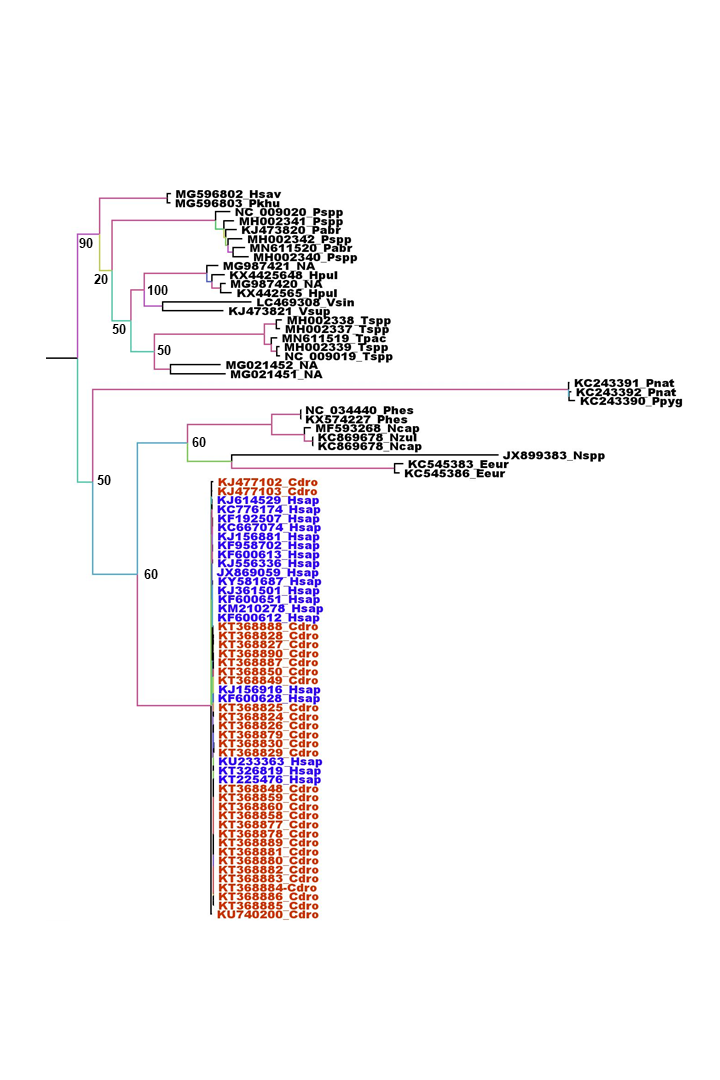

Supplement: Supplementary Figure 5 — Phylogenetic analysis of Merbecoviruses spike genes. The alignment of the full spike genes was performed with MUSCLE from the SeaView package (Gouy et al., 2010). The tree was built using the maximum likelihood method under the GTR model with 500 repeats. The tree was rooted using the spike gene sequence of a Sarbecovirus from Manis javanica (MT040335) as outgroup. Deep blue: spike gene sequences from human MERS-CoV. Red: spike gene sequences from dromedaries MERS-CoV. Sample names are built with the GenBank accession number followed by a four-letter code identifying the species. The species codes are the following: Cdro, Camelus dromedarius; Eeur, Erinaceus europaeus; Hpul, Hypsugo pulveratus; Hsap, Homo sapiens; Hsav, Hypsugo savii; Ncap, Neoromicia capensis; Nspp, Nycteris unidentified species; Nzul, Neoromicia zuluensis (synonymous to Neoromicia capensis); Pabr, Pipistrellus abramus; Phes, Pipistrellus hesperidus; Pkuh, Pipistrellus kuhlii; Pnat, Pipistrellus nathusii; Ppyg, Pipistrellus pygmaeus; Pspp, Pipistrellus unidentified species; Tpac, Tylonycteris pachypus; Tspp, Tylonycteris unidentified species; Vsin, Vespertilio sinensis; Vsup, Vespertilio superans; NA, Not available. [file Image_5.TIF]

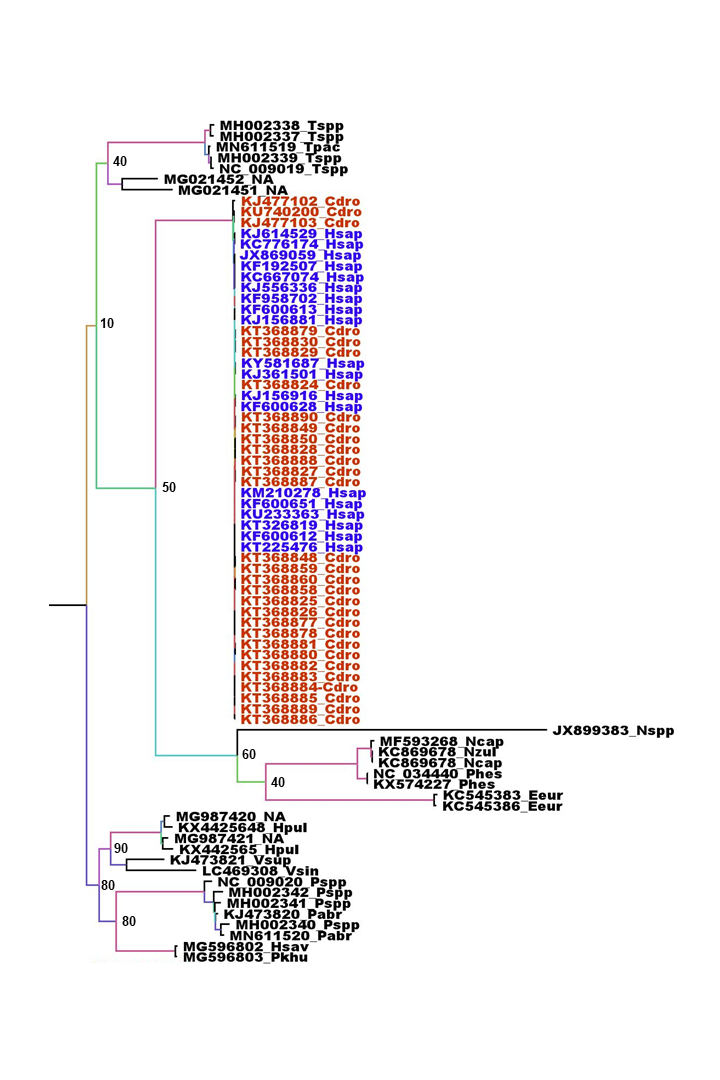

Supplement: Supplementary Figure 6 — Distribution of Merbecoviruses spike protein sequences. The alignment of the translated full spike genes was performed with MUSCLE from the SeaView package (Gouy et al., 2010). The tree was built using the maximum likelihood method under the LG model with 500 repeats. The tree was rooted using the spike protein sequence of a Sarbecovirus from Manis javanica (MT040335) as outgroup. Deep blue: spike protein sequences from human MERS-CoV. Red: spike protein sequences from dromedaries MERS-CoV. Sample names are built with the GenBank accession number followed by a four-letter code identifying the species. The species codesare the following: Cdro, Camelus dromedarius; Eeur, Erinaceus europaeus; Hpul, Hypsugo pulveratus; Hsap, Homo sapiens; Hsav, Hypsugo savii; Ncap, Neoromicia capensis; Nspp, Nycteris unidentified species; Nzul, Neoromicia zuluensis (synonymous to Neoromicia capensis); Pabr, Pipistrellus abramus; Phes, Pipistrellus hesperidus; Pkuh, Pipistrellus kuhliiv; Pnat, Pipistrellus nathusii; Ppyg, Pipistrellus pygmaeus; Pspp, Pipistrellus unidentified species; Tpac, Tylonycteris pachypus; Tspp, Tylonycteris unidentified species; Vsin, Vespertilio sinensis; Vsup, Vespertilio superans; NA, Not available. [file Image_6.TIF]
