# Supplementary material for: Emergence of Bat-Related Betacoronaviruses: Hazard and Risks
Source: Front Microbiol. 2021 Mar 15;12:591535. doi: 10.3389/fmicb.2021.591535 (PMC8005542; doi:10.3389/fmicb.2021.591535)
Supplement: Supplementary file 7 [file Data_Sheet_1.docx]

**Supplementary data: Comparison of the DPP4 protein sequences in 16 different species.**

All DPP4 sequences (from GenBank and NCBI) were compared using the Clustal Omega multiple sequence alignment (EMBL-EBI bioinformatic tool; Copyright © EMBL 2020). The accession numbers for the selected sequences are listed hereafter: *Homo sapiens* (Hsap, GenBank: AAH13329.2); *Macaca mulatta* (Mmul, NCBI Reference Sequence: NP_001034279.2); *Camelus dromedarius* (Cdro, GenBank: AIG55259.1); *Camelus bactrianus* (Cbac, NCBI Reference Sequence: XP_010969750.1); *Camelus ferus* (Cfer, NCBI Reference Sequence: XP_006176870.1); *Sus scrofa* (Sscr, NCBI Reference Sequence: NP_999422.1); *Mustela putorius furo* (Mput, GenBank: ABC72084.1); *Felis catus* (Fcat, NCBI Reference Sequence: NP_001009838.1); *Rattus rattus* (Rrat, NCBI Reference Sequence: XP_03275977.1); *Mus musculus* (Mmus, GenBank: CAA41274.1); *Pelodiscus sinensis* (Psin, NCBI Reference Sequence: XP_006114903.1); *Manis javanica* (Mjav, NCBI Reference Sequence: XP_017519864.1); *Rhinolophus sinicus* (Rsin, GenBank: AZO92863.1); *Rhinolophus ferrumequinum* (Rfer, GenBank: AXB27025.1); *Pipistrellus abramus* (Pabr, GenBank: AZO922861.1); *Tylonycteris pachypus* (Tpac, GenBank: AZO92860.1). The phylogenetic tree was generated according to the Unweighted pair group method with arithmetic mean (UPGMA)/Neighbor joining method and the multiple sequence alignment of DPP4 was generated using the CLUSTAL O (1.2.4) multiple sequence alignment program (Results for job clustalo-E20200421-084358-0979-47413847-p2m). Within the amino acid sequences of DPP4 important for MERS-CoV spike binding the conserved amino acids are highlighted in yellow, those critical for MERS-CoV-DPP4 binding are highlighted in red

**UPGMA tree of the DDPA protein sequences**


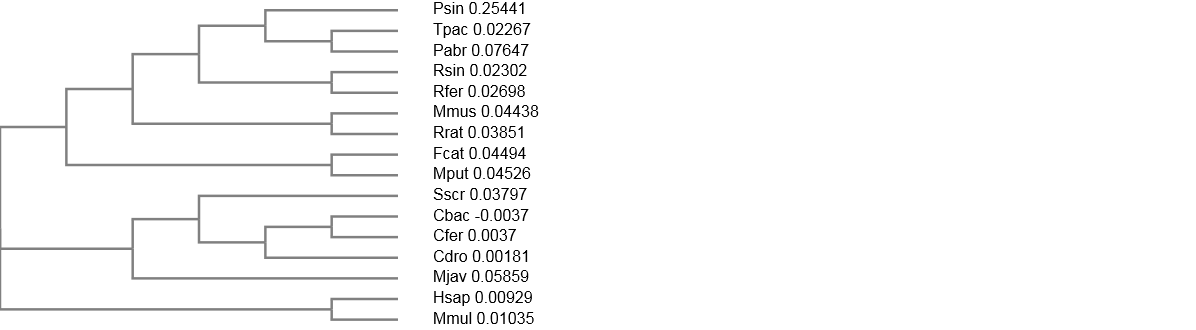


**CLUSTAL O (1.2.4) multiple sequence alignment of the DPP4 protein sequences**

**Psin MKTAVKYLLGLLAVAVIVTAIAVPIALLAKNAG-AKSDSRRTFTLEDYLNGEIKYKTHNL 59**

**Tpac MKTPWKVLLGLLGTAALVTIITVPAVLLSQGND-ATADSRRTYTLTDYLKSTIRTRNYNL 59**

**Pabr ------------------------------------------------------------ 0**

**Mmus MKTPWKVLLGLLGVAALVTIITVPIVLLSKD--EAAADSRRTYSLADYLKSTFRVKSYSL 58**

**Rrat MKTPWKVLLGLLGVAALVTIITVPVVLLNKD--EAAADSRRTYTLADYLKNTFRVKSYSL 58**

**Rsin ----------------------VPVVLLNKDTDDSTADSRRTYTLTDYLKSTFRTKAYNL 38**

**Rfer MKTPWRVLLGLLGAAALVTIITVPVVLLNKGTDDSTADSRRTYTLTDYLKNTFRTKAYSL 60**

**Fcat MKTPWKVLLGLLGLAALITIITVPVVLLNKGND-AAADSRRTYTLTDYLKNTFRVKFYSL 59**

**Mput MKTPWRVLLGLLGLAALVTVITVPAVLLNKGND-ATADSRRTYTLTDYLKNTFRMKFYSL 59**

**Sscr MKTPWKVLLGLLGIAALVTVITVPVVLLNKGTDDAAADSRRTYTLTDYLKSTFRVKFYTL 60**

**Cbac MKTPWKVLLGLLGIAALVTVITVPVVLLNKGTDDATADSRRTYTLTDYLKNTFRLKVYTL 60**

**Cdro MKTPWRVLLGLLGIAALVTLITVPAVLLSKGTDDATADSRRTYTLTDYLKNTFRLKVYTL 60**

**Cfer MKTPWKVLLGLLGIAALVTVITVPVVLLNKGTDDATADSRRTYTLTDYLKNTFRLKVYTL 60**

**Mjav MKTLWKVLVGLLGAAALVTVITVPVVLLTRGTDDTTADSRRTYTLTDYLKNTFRVKFYSL 60**

**Hsap --TPWKVLLGLLGAAALVTIITVPVVLLNKGTDDATADSRKTYTLTDYLKNTYRLKLYSL 58**

**Mmul MKTAWKVLLGLLGAAALVTIITVPVVLLNKGTDDATADSRKTYTLTDYLKNTYRLKLYSL 60**

**Psin QWISGNEYLHKTADNDIVLINADDQTLSEVLKNTTLATYGVT--TAILSPDQKFALFQYN 117**

**Tpac WWISDHEYLYRQE-NNILLFNADLGNSSTFLENSTFDQFGHSINDYSVSPDRQFVLLEYN 118**

**Pabr ------------------------------------------------------------ 0**

**Mmus WWVSDFEYLYKQE-NNILLLNAEHGNSSIFLENSTFESFGY----HSVSPDRLFVLLEYN 113**

**Rrat RWVSDSEYLYKQE-NNILLFNAEHGNSSIFLENSTFEIFGDSISDYSVSPDRLFILLEYN 117**

**Rsin RWVSDHEYLYKQE-NNILLFNAEYGNSSFFLENSTFDQFGHSINDYSVSPDGQFVLLEYN 97**

**Rfer RWVSDHEYLYKQE-NNILLFNAEYGNSSILLENSSFDQFGHSINDYSVSPDGQFVLLEYN 119**

**Fcat RWVSDHDYLYKQD-NNILLFNAEYGNSSIFLENSTFDEFEHSINDYSVSPDGQFILLEYN 118**

**Mput SWISDQEYLYKQE-NNILLFNAEYGNSSIFLENSTFEEFEHTINDYLVSPDGQFILLEYN 118**

**Sscr QWISDHEYLYKQE-NNILLFNAEYGNSSIFLENSTFDELGYSTNDYSVSPDRQFILFEYN 119**

**Cbac QWVSDHEYLYRQE-NNILLFNAEYGNSSIFLENSTFDEFGHSINDYSVSPDRQYILFEYN 119**

**Cdro QWVSDHEYLYRQE-NNILLFNAEYGNSSIFLENSTFDEFGHSINDYSVSPDRQYILFEYN 119**

**Cfer QWVSDHEYLYRQE-NNILLFNAEYGNSSIFLENSTFDEFGHSINDYSVSPDRQYILFEYN 119**

**Mjav RWVSDHEYLYKQE-NNILLFNAEYGNSSIFLENSTFDEFGHSINDYSVSPDGQFILFEYN 119**

**Hsap RWISDHEYLYKQE-NNILVFNAEYGNSSVFLENSTFDEFGHSINDYSISPDGQFILLEYN 117**

**Mmul RWISDHEYLYKQE-NNILVFNAEYGNSSVFLENSTFDEFGHSINDYSISPDGQFILLEYN 119**

**Psin YVKLWRHSYTASYHIYDIQSRSLVTENLLPNDTQYISWSPVGHKLAYVWNNNVYIKESPG 177**

**Tpac YEKKWRYSYTASYDIYDLNKRQLITEERIPNDTQLISWSPEGHKLAYVWNNDIYIKNDPN 178**

**Pabr ---------------------------------------------------DIYIKNDPS 9**

**Mmus YVKQWRHSYTASYNIYDVNKRQLITEEKIPNNTQWITWSPEGHKLAYVWKNDIYVKVEPH 173**

**Rrat YVKQWRHSYTASYSIYDLSKRQLITEEKIPNNTQWITWSQEGHKLAYVWKNDIYVKIEPH 177**

**Rsin YVKKWRHSYTASYDIYDLNKRQLITEERIPNNTQLITWSPEGHKLAYVWNNDIYIKNEPN 157**

**Rfer YVKKWRHSYTASYDIYDLNKRQLITEERIPNDTQLITWSPEGHKLAYVWNNDIYIKNEPN 179**

**Fcat YVKQWRHSYTASYDIYDLNKRQLITEEKIPNNTQWITWSPEGHKLAYVWKNDVYVKNEPN 178**

**Mput YMKQWRHSYTASYDIYDLAKRRLITEDKIPDNTQWITWSPEGHKLAYVWNNDIYVKNEPN 178**

**Sscr YVKQWRHSYTASYDIYDLNKRQLITEERIPNNTQWITWSPVGHKLAYVWNNDIYVKNEPN 179**

**Cbac YVKQWRHSYTASYDIYDLKKRQLITEERIPNNTQWITWSPVGHKLAYVWNNDIYVKSEPN 179**

**Cdro YVKQWRHSYTASYDIYDLKKRQLITEERIPNNTQWITWSPVGHKLAYVWNNDIYVKSEPN 179**

**Cfer YVKQWRHSYTASYDIYDLKKRQLITEERIPNNTQWITWSPVGHKLAYVWNNDIYVKSEPN 179**

**Mjav YVKKWRHSYTASYDIYDLHKRQLITEERIPNNTQWITWSPVGHKLAYVWHNDIYIKKEPN 179**

**Hsap YVKQWRHSYTASYDIYDLNKRQLITEERIPNNTQWVTWSPVGHKLAYVWNNDIYVKIEPN 177**

**Mmul YVKQWRHSYTASYDIYDLNKRQLITEERIPNNTQWVTWSPVGHKLAYVWNNDIYVKIEPN 179**

**::*:* .***

**Psin AASVPITTNGEENKIFNGIADWVYEEEMFGTHSALWWSPNASFLAYAEFNDTEVPVMEYS 237**

**Tpac SPSQRVTDNGREDAISNGITDWVYEEEIFNTHSALWWSPNGTFLAYAQFNDTEVPRIEYS 238**

**Pabr SPAQRVTHDGREDAISNGITDWVYEEEIFNTHSALWWSPNGTFLAYARFDDIQVPRIEYS 69**

**Mmus LPSHRITSTGEENVIYNGITDWVYEEEVFGAYSALWWSPNNTFLAYAQFNDTGVPLIEYS 233**

**Rrat LPSHRITSTGKENVIFNGINDWVYEEEIFGAYSALWWSPNGTFLAYAQFNDTGVPLIEYS 237**

**Rsin SASQRITSNGKEDVINNGITDWVYEEEIFSTHSALWWSPNSTFLAYAQFNDTQVPPIEFS 217**

**Rfer SASQRITWNGKEDVI-NGIPDWVYEEEIFSTHSALWWSPTGTFLAYAQFNDTEVPPIEFS 238**

**Fcat SSSHRITWTGEENAIYNGIADWVYEEEIFSAYSALWWSPKGTFLAYAQFNDTQVPLIEYS 238**

**Mput STSQRITWTGKENVISNGITDWVYEEEIFSAYSALWWSPKGTFLAYAQFNDTEVPLIEYS 238**

**Sscr LSSQRITWTGKENVIYNGVTDWVYEEEVFSAYSALWWSPNGTFLAYAQFNDTEVPLIEYS 239**

**Cbac LPSQRITWTGKKDVIYNGITDWVYEEEVFSAYSALWWSPNGTFLAYAQFNDTEVPLIEYS 239**

**Cdro LPSQRITWTGKKDVIYNGITDWVYEEEVFSAYSALWWSPNGTFLAYAQFNDTEVPLIEYS 239**

**Cfer LPSQRITWTGKKDVIYNGITDWVYEEEVFSAYSALWWSPNGTFLAYAQFNDTEVPLIEYS 239**

**Mjav LQSQRITWTGKEDVIYNGITDWVYEEEVFSAYSALWWSPNGTFLAYAQFNDTEVPLIEYS 239**

**Hsap LPSYRITWTGKEDIIYNGITDWVYEEEVFSAYSALWWSPNGTFLAYAQFNDTEVPLIEYS 237**

**Mmul LPSHRITSTGKEDMIYNGITDWVYEEEVFSAYSALWWSPNGTFLAYAQFNDTEVPLIEYS 239**

**: :* *.:: * **: *******:*.::*******. :*****.*:* ** :*:***

**Psin FYAEDTLQYPKTIRLPYPKAGATNPTVKLFVVNTQSPST---VNTSEIIAPASIISGDHY 294**

**Tpac VYLDESLQYPKTVHIPYPKAGAENPTVKLYVVNTDSLTN---LVPKQITAPASVLKGDHY 295**

**Pabr VYLDESLQYPKTVHLPYPKAGAENPTVKFYIVNTDNLTN---LVPVQITAPASVLIGDHY 126**

**Mmus FYSDESLQYPKTVWIPYPKAGAVNPTVKFFIVNIDSLSSSSSAAPIQIPAPASVARGDHY 293**

**Rrat FYSDESLQYPKTVWIPYPKAGAVNPTVKFFIVNTDSLSSTTTTIPMQITAPASVITGDHY 297**

**Rsin VYFDESLQYPKTVHLPYPKAGAMNPTVKFFVVNTDNLTN---AVPKQIVAPASILIGDHY 274**

**Rfer VYFDESLQYPKTMHIPYPKAGAMNPTVKFFVVNTDNLTS---AVPKQIVAPASMLIGDHY 295**

**Fcat FYSDESLQYPMTMRIPYPKAGAANPTVKLFVIKTDNLNPNTNATSVEITPPAAMLTGDYY 298**

**Mput FYSDESLQYPKTVRIPYPKAGAVNPTVKLFVVKINNLDPNTNATSVEITPPDAISTGDYY 298**

**Sscr FYSDESLQYPKTVRIPYPKAGAENPTVKFFVVDTRTLSPNASVTSYQIVPPASVLIGDHY 299**

**Cbac FYSDESLQYPKTVRIPYPKAGAVNPTVKFFVVDTSTLSPNVNATSRQIVPPASVLIGDHY 299**

**Cdro FYSDESLQYPKTVRIPYPKAGAVNPTVKFFVVDTSTLSPNVNATSRQIVPPASVLIGDHY 299**

**Cfer FYSDESLQYPKTVRIPYPKAGAVNPTVKFFVVDTSTLSPNVNATSRQIVPPASVLIGDHY 299**

**Mjav FYSDESLQYPKTMQVPYPKAGAANPTVKLFVVNTDSLNSTANATSVQVVPPASVLIGDHY 299**

**Hsap FYSDESLQYPKTVRVPYPKAGAVNPTVKFFVVNTDSLSSVTNATSIQITAPASMLIGDHY 297**

**Mmul FYSDESLQYPKTVQVPYPKAGAVNPTVKFFVVNTDSLSSATNATSIQITAPASMLIGDHY 299**

**.* :::**** *: :******* *****::::. . :: * :: **:***

**Psin LSVVTWVTDERICLQWLRRIQNFSVLAVCDFMRD-SGTWQCPKDKQHTEESKTGWIGTFQ 353**

**Tpac LCDVTWATQERISLQWLRRIQNYSIIDICDYDESHDSRWNCSVPRQHIETSTTGWVGRFK 355**

**Pabr LCDVTWATKERISLQWLRRIQNYAILDICDYDGA-YSTWNCSVSRQHIETSTTGWVGRFK 185**

**Mmus LCDVVWATEERISLQWLRRIQNYSVMAICDYDKI-NLTWNCPSEQQHVEMSTTGWVGRFR 352**

**Rrat LCDVAWVSEDRISLQWLRRIQNYSVMAICDYDKT-NLIWNCPMTQEHIETSATGWVGRFR 356**

**Rsin LCDVTWVTKERISLQWLRRIQNYSVMDICDYDEA-NEGWTCSVARQHIEMSTTGWVGRFK 333**

**Rfer LCDVTWVTKERISLQWLRRIQNYSIMDICDYDEF-NDRWTCLVGRQHIEMSTTGWVGRFK 354**

**Fcat LCDVTWANEERISLQWLRRIQNYSVMDIRDYNNS-TGKWISSAAQEHIEMSTTGWVGRFR 357**

**Mput LCDVTWVNEERISLQWLRRIQNYSVMNICDYNND-TNSWRKPEAQEHTEMSTTGWVGRFR 357**

**Sscr LCGVTWVTEERISLQWIRRAQNYSIIDICDYDES-TGRWISSVARQHIEISTTGWVGRFR 358**

**Cbac LCGVTWVTEKRISLQWIRRIQNYSIMDVCDYDES-TGRWASSVGRQHIETSTTGWVGRFR 358**

**Cdro LCGVTWVTEKRISLQWIRRIQNYSIMDVCDYDES-TGRWASSVGRQHIETSTTGWVGRFR 358**

**Cfer LCGVTWVTEKRISLQWIRRIQNYSIMDVCDYDES-TGRWASSVGRQHIETSTTGWVGRFR 358**

**Mjav LCDVTWVNEERISLQWLRRIQNYSVLAICDYDKP-TGRWASHVRQQHIESSTTGWVGRFK 358**

**Hsap LCDVTWATQERISLQWLRRIQNYSVMDICDYDES-SGRWNCLVARQHIEMSTTGWVGRFR 356**

**Mmul LCDVTWATQERISLQWLRRIQNYSVMDICDYDES-SGRWNCLVARQHIETSTTGWVGRFR 358**

***. *.*....**.***:** **:::: : *: * ::* * * ***:* *:**

**Psin PSDPYFAPDNVGYYKILSNTEGYKHIHYINGSG----SRKPITNGKWEVISIEAVTSDFI 409**

**Tpac PAEPHFTADGNSFYKIMSNSEGYKHICHFQVDN---QKCTFITNGTWEVIKIEALTNNYL 412**

**Pabr PAEPHFTADGNSFYKIMSNREGYKHICHFQADN---DVCTFITDGTWEVIKIEALTNNYL 242**

**Mmus PAEPHFTSDGSSFYKIISDKDGYKHICHFPKDK---KDCTFITKGAWEVISIEALTSDYL 409**

**Rrat PAEPHFTSDGSSFYKIVSDKDGYKHICQFQKDRKPEQDCTFITKGAWEVISIEALTSDYL 416**

**Rsin PSEPHFTSDGKSFYKIISDKEGYKHICFFQIDT---QNCTFITKGAWEVISIAALSSDYL 390**

**Rfer PSKPRFTSDGKSVYKIISNKEGYKHICFFQVDT---ENCTFITKGAWEVIGIAALTSDYL 411**

**Fcat PAEPHFTSDGRNFYKIISNEDGYKHICRFQIDK---KDCTFITKGAWEVIGIEALTTDYL 414**

**Mput PSEPHFTSDGKSFYKIISNQDGYKHICLFQIDK---QDCTFITKGAWEVIGIEALTSDYL 414**

**Sscr PAEPHFTSDGNSFYKIISNEEGYKHICHFQTDK---SNCTFITKGAWEVIGIEALTSDYL 415**

**Cbac PAEPHFTSDGSSFYKIISNEEGYKHICHFQTDK---RNCTFITKGAWEVIGIEALTRDYL 415**

**Cdro PAEPHFTSDGSSFYKIISNEEGYKHICHFQTDK---RNCTFITKGAWEVIGIEALTRDYL 415**

**Cfer PAEPHFTSDGSSFYKIISNEEGYKHICHFQTDK---RNCTFITKGAWEVIGIEALTRDYL 415**

**Mjav PSEPHFTSDGNSFYKIISNKEGYKHICHFQVDR---ENCTFITKGVWEVIGIEALTSDDL 415**

**Hsap PSEPHFTLDGNSFYKIISNEEGYRHICYFQIDK---KDCTFITKGTWEVIGIEALTSDYL 413**

**Mmul PSEPHFTSDGNSFYKIISNEEGYRHICYFQINK---KNCTFITKGAWEVIGIEALTSDYL 415**

***:.* *: *. . ***:*: :**:** : . . **.* **** * *:: : :**

**Psin YYISNEFDGKPGGRNLYKVSVGNSPTTPKCVSCDWDKDRCRYYSASFSSDAKYYLLNCYG 469**

**Tpac YYISNEYKGMPGGRNLYKIQLNNN-TNVACLSCELDPERCQYYSASFSKGAKYYQLRCSG 471**

**Pabr YYISNEYKGMPGGRNLYKIQLNNI-TNVACVSCELFPERCQYYSASFSKGGKYYQLRCSG 301**

**Mmus YYISNQYKEMPGGRNLYKIQLTDH-TNVKCLSCDLNPERCQYYAVSFSKEAKYYQLGCWG 468**

**Rrat YYISNEYKEMPGGRNLYKIQLTDL-TNKKCLSCDLSPERCQYYSVSFSKEAKYYQLGCRG 475**

**Rsin YYISNEYKGMPGGRNLYKIQLNDY-TKVTCLSCELNPKECQYYSASFSKEAKYYQLRCSG 449**

**Rfer YYISNEYKGMPGGRNLYKIQLNDY-TKVICLSCELNPKECQYYSASFSKEAKYYQLICSG 470**

**Fcat YYISNEYKGMPGGRNLYKIQLNDY-TKVACLSCELKPERCQYYSVSFSKEAKYYQLRCSG 473**

**Mput YYISNEYKGMPGGRNLYKIQLSNY-TKVTCLSCELNPERCQYYSVSFSKEAKYYQLRCSG 473**

**Sscr YYISNEHKGMPGGRNLYRIQLNDY-TKVTCLSCELNPERCQYYSASFSNKAKYYQLRCFG 474**

**Cbac ---------MPGGRNLYKVQLNDY-TKVTCLTCELDPERCQYYSASFSKEAKYYQLRCSG 465**

**Cdro YYISNEHKGMPGGRNLYKVQLNDY-TKVTCLTCELDPERCQYYSASFSKEAKYYQLRCSG 474**

**Cfer YYISNEHKGMPGGRNLYKVQLNDY-TKVTCLTCELDPERCQYYSASFSKEAKYYQLRCSG 474**

**Mjav YYISNEYKGMPGGRNLYKIQLNDH-TKVTCLSCELNTERCQYYSVSFSKEAKYYQLRCSG 474**

**Hsap YYISNEYKGMPGGRNLYKIQLSDY-TKVTCLSCELNPERCQYYSVSFSKEAKYYQLRCSG 472**

**Mmul YYISNEYKGMPGGRNLYKIQLSDY-TKVTCLSCELNPERCQYYSVSFSKEAKYYQLRCSG 474**

*********::.: : *. *::*: ..*:**:.***. .*** * * ***

**Psin PGLPTSMLFRSSDDKVNRTLENNTDLNSTLKNIQMPSKRIDTISLNGYTLWYQMILPPHF 529**

**Tpac PQLPRYSLHNSSDDKELRLLENNTALYEALGNIQMPRKTLDFIYMHGMKFWYQMILPPHF 531**

**Pabr PQLPRYSVHSSSNDTELRLLENNTDLYATLRNIKMPRKTLDFIQLHGTKFWYQMILPPHF 361**

**Mmus PGLPLYTLHRSTDHKELRVLEDNSALDRMLQDVQMPSKKLDFIVLNETRFWYQMILPPHF 528**

**Rrat PGLPLYTLHRSTDQKELRVLEDNSALDKMLQDVQMPSKKLDFIVLNETRFWYQMILPPHF 535**

**Rsin PGLPRYTMHSSSNDKELRVLEDNSALAKMLQDVQMPTKELNFIILNETKFWYQMILPPHF 509**

**Rfer PGLPRYTLHSSSNDKELRVLENNSALAKMLQDVQMPTKELNFIILNETKFWYQMILPPHF 530**

**Fcat PGLPLYTLHRSSNDEELRVLEDNSALDKMLQEVQMPSKKLDFIILNETKFWYQMILPPHF 533**

**Mput PGLPLYTLHRSSDDKELRVLEDNSALNKMLQDVQMPSKKLDFIILNQTKFWYQMILPPHF 533**

**Sscr PGLPLYTLHSSSSDKELRVLEDNSALDKMLQDVQMPSKKLDVINLHGTKFWYQMILPPHF 534**

**Cbac PGLPLYTLHSSSSDKELRVLENNSALENMLQEVQMPTKKLDFINMHETKFWYQMILPPHF 525**

**Cdro PGLPLYTLHSSSSDKELRVLENNSALENMLQEVQMPTKKLDFINMHETKFWYQMILPPHF 534**

**Cfer PGLPLYTLHSSSSDKELRVLENNSALENMLQEVQMPTKKLDFINMHETKFWYQMILPPHF 534**

**Mjav PGLPLYTLHSSSNDKELRVLEDNSALDKMLQDVQMPSKKLDFIIWNGIKLWYQMILPPHF 534**

**Hsap PGLPLYTLHSSVNDKGLRVLEDNSALDKMLQNVQMPSKKLDFIILNETKFWYQMILPPHF 532**

**Mmul PGLPLYTLHSSVNDKGPRVLEDNSALDKMLQNVQMPSKTLDFIILNETKFWYQMILPPHF 534**

*** ** :. * .. * **:*: * * :::** * :: * : :************

**Psin DSSKKYPLLLDVYAGPCSQKVDYAFRINWATYLASTEQIIVASFDGRGSGYQGDEIMHAI 589**

**Tpac DKSKKYPLLIDVYAGPCSQKADATFTLNWATYLASTENIIVASFDGRGSGYQGDKIMHAI 591**

**Pabr DKSKKYPLLIDVYGGPCSQKADATFTLNWATYLASTENIIVASFDGRGSGYQGDKIMHAI 421**

**Mmus DKSKKYPLLLDVYAGPCSQKADASFRLNWATYLASTENIIVASFDGRGSGYQGDKIMHAI 588**

**Rrat DKSKKYPLLIDVYAGPCSQKADAAFRLNWATYLASTENIIVASFDGRGSGYQGDKIMHAI 595**

**Rsin DKSKKYPLLIDVYAGPCSQKADATFTLNWATYLASTENIIVASFDGRGSGYQGDKIMHAI 569**

**Rfer DKSKKYPLLIDVYAGPCSQKADATFTLNWATYLASTENIIVASFDGRGSGYQGDKIMHAI 590**

**Fcat DTSKKYPLLIDVYAGPCSQKADAIFRLNWATYLASTENIIVASFDGRGSGYQGDKIMHAV 593**

**Mput DTSKKYPLLIDVYAGPCSQKADAIFRLNWATYLASTENIIVASFDGRGSGYQGDKIMHAV 593**

**Sscr DKSKKYPLLIEVYAGPCSQKVDTVFRLSWATYLASTENIIVASFDGRGSGYQGDKIMHAI 594**

**Cbac DKSKKYPLLIDVYAGPCSQKADTIFRLNWATYLASTENIIVASFDGRGSGYQGDKIMHAI 585**

**Cdro DKSKKYPLLIDVYAGPCSQKADTIFRLNWATYLASTENIIVASFDGRGSGYQGDKIMHAI 594**

**Cfer DKSKKYPLLIDVYAGPCSQKADTIFRLNWATYLASTENIIVASFDGRGSGYQGDKIMHAI 594**

**Mjav DKLKKYPLLIDVYAGPCSQKADTVFRLNWATYLASTEKIIVASFDGRGSGYQGDKILHAI 594**

**Hsap DKSKKYPLLLDVYAGPCSQKADTVFRLNWATYLASTENIIVASFDGRGSGYQGDKIMHAI 592**

**Mmul DKSKKYPLLLDVYAGPCSQKADAVFRLNWATYLASTENIIVASFDGRGSGYQGDKIMHAI 594**

***. ******::**.******.* * :.*********:****************:*:**:**

**Psin NRRLGTYEVEDQISAARKFSEMSFVDKNRIAIWGWSYGGYVTSMVLGSGSGVFKCGIAVA 649**

**Tpac NRRLGTLEVEDQIEAARQFSKMGFVDDKRIAIWGWSYGGYVTSMVLGAGSGVFKCGIAVA 651**

**Pabr YRRLGTFEVEDQIEAARQFSKMGFVDDKRIAIWGWSYGGYVTS----------------- 464**

**Mmus NRRLGTLEVEDQIEAARQFVKMGFVDSKRVAIWGWSYGGYVTSMVLGSGSGVFKCGIAVA 648**

**Rrat NKRLGTLEVEDQIEAARQFLKMGFVDSKRVAIWGWSYGGYVTSMVLGSGSGVFKCGIAVA 655**

**Rsin NRRLGTLEVQDQIEAARQFSKMGFVDHERIAIWGWSYGGYVTSMVLGAGSHVFKCGIAVA 629**

**Rfer NRRLGTLEVQDQIEAARQFSKMGFVDHERIAIWGWSYGGYVASMVLGAGSHVFKCGIAVA 650**

**Fcat NRRLGTFEVEDQIEAARQFSKMGFVDDKRIAIWGWSYGGYVTSMVLGAGSGVFKCGIAVA 653**

**Mput NRRLGTFEVEDQIEAARQFSKMGFVDDKRIAIWGWSYGGYVTSMVLGAGSGVFKCGIAVA 653**

**Sscr NRRLGTFEVEDQIEATRQFSKMGFVDDKRIAIWGWSYGGYVTSMVLGAGSGVFKCGIAVA 654**

**Cbac NRRLGTFEVEDQIEATRQFSKMGFVDDKRIAIWGWSYGGYVTSMVLGAGSGVFKCGIAVA 645**

**Cdro NRRLGTFEVEDQIEATRQFSKMGFVDDKRIAIWGWSYGGYVTSMVLGAGSGVFKCGIAVA 654**

**Cfer NRRLGTFEVEDQIEATRQFSKMGFVDDKRIAIWGWSYGGYVTSMVLGAGSGVFKCGIAVA 654**

**Mjav NRRLGTLEVEDQIEAARQFTNMGFVDEKRIAIWGWSYGGYVTSMVLGAGSGVFKCGIAVA 654**

**Hsap NRRLGTFEVEDQIEAARQFSKMGFVDNKRIAIWGWSYGGYVTSMVLGSGSGVFKCGIAVA 652**

**Mmul NRRLGTFEVEDQIEAARQFSKMGFVDNKRIAIWGWSYGGYVTSMVLGSGSGVFKCGIAVA 654**

**:**** **:***.*:*:* :*.*** :*:***********:***

**Psin PVSRWQYYDSIYTERYMGLPVASDNLKNYESSTVMARAKNFTQVEYLLIHGTADDNVHFQ 709**

**Tpac PVSAWEFYDSVYTERYMGLPTSEDNLDHYKNSTVMSRAEHFKQVEYLLIHGTADDNVHFQ 711**

**Pabr ------------------------------------------------------------ 464**

**Mmus PVSRWEYYDSVYTERYMGLPIPEDNLDHYRNSTVMSRAEHFKQVEYLLIHGTADDNVHFQ 708**

**Rrat PVSRWEYYDSVYTERYMGLPTPEDNLDHYRNSTVMSRAENFKQVEYLLIHGTADDNVHFQ 715**

**Rsin PVSAWEFYDSVYTERYMGLPTVEDNLGHYKNSTVMSRAENFKEVEYLLIHGTADDNVHFQ 689**

**Rfer PVSAWEFYDSVYTERYMGLPTVEDNLGHYKNSTVMSRAENFKEVAYLLIHGTADDNVHFQ 710**

**Fcat PVSRWEYYDSVYTERYMGLPTPQDNLDYYKNSTVMSRAENFKQVEYLLIHGTADDNVHFQ 713**

**Mput PVSRWEYYDSVYTERYMGLPTPEDNLDYYRNSTVMSRAENFKQVEYLLIHGTADDNVHFQ 713**

**Sscr PVSKWEYYDSVYTERYMGLPTPEDNLDYYRNSTVMSRAENFKQVEYLLIHGTADDNVHFQ 714**

**Cbac PVSKWEYYDSVYTERYMGLPTPQDNLDYYRNSTVMSRAENFKQVEYLLIHGTADDNVHFQ 705**

**Cdro PVSKWEYYDSVYTERYMGLPTPQDNLDYYRNSTVMSRAENFKQVEYLLIHGTADDNVHFQ 714**

**Cfer PVSKWEYYDSVYTERYMGLPTPQDNLDYYRNSTVMSRAENFKQVEY-------------- 700**

**Mjav PVSRWEYYDSVYTERYMGLPTPEDNLEHYRNSTVMSRAENFKQVEYLLIHGTADDNVHFQ 714**

**Hsap PVSRWEYYDSVYTERYMGLPTPEDNLDHYRNSTVMSRAENFKQVEYLLIHGTADDNVHFQ 712**

**Mmul PVSRWEYYDSVYTERYMGLPTPEDNLDHYRNSTVMSRAENFKQVEYLLIHGTADDNVHFQ 714**

**Psin QAAQISKALVDAQVDFQAMWYTDKDHGIGG-LAHSHIYTHMSHFIKQCFSLP 760**

**Tpac QSAQITKALVDAGVDFQAMWYTDEDHGIASNTAHQHIYTHMTHFLKQCFSLP 763**

**Pabr ---------------------------------------------------- 464**

**Mmus QSAQISKALVDAGVDFQAMWYTDEDHGIASSTAHQHIYSHMSHFLQQCFSLH 760**

**Rrat QSAQISKALVDAGVDFQAMWYTDEDHGIASSTAHQHIYSHMSHFLQQCFSLR 767**

**Rsin QSAHISKALVDAGVDFQAMWYTDEDHGIGTSTAHQHIYTHMSHFIKQCFSLP 741**

**Rfer QSAHISRALVDAGVDFQAMWYTDEDHGIGTSTAHQHIYTHMSHFIKQCFSLP 762**

**Fcat QSAQISKALVDAGVDFQAMWYTDEDHGIASGPAHQHIYTHMSHFIKQCFSLP 765**

**Mput QSAQISKALVDAGVDFQAMWYTDEDHGIASSTAHQHIYTHMSHFIKQCFSLP 765**

**Sscr QSAQLSKALVDAGVDFQTMWYTDEDHGIASNMAHQHIYTHMSHFLKQCFSLP 766**

**Cbac QSAQISKALVDAGVDFQTMWYTDEDHGIASSTAHQHIYTHMSHFLKQCFSLP 757**

**Cdro QSAQISKALVDAGVDFQTMWYTDEDHGIASSTAHQHIYTHMSHFLKQCFSLP 766**

**Cfer ---------------------------------------------------- 700**

**Mjav QSAQISKALVDAGVDFQAMWYTDEDHGIASSTAHQHIYTHMSHFIKQCFSLP 766**

**Hsap QSAQISKALVDVGVDFQAMWYTDEDHGIASSTAHQHIYTHMSHFIKQCFSLP 764**

**Mmul QSAQISKALVDAGVDFQAMWYTDEDHGIASSTAHQHIYTHMSHFIKQCFSLP 766**
